# A Survey of *Helicobacter pylori* Antibiotic-Resistant Genotypes and Strain Lineages by Whole-Genome Sequencing in China

**DOI:** 10.1128/aac.02188-21

**Published:** 2022-06-02

**Authors:** Yan Zhou, Zishao Zhong, Shengjuan Hu, Jing Wang, Yanhong Deng, Ximei Li, Xianmei Chen, Xue Li, Yuanyuan Tang, Xiaofei Li, Qian Hao, Jun Liu, Tian Sang, Yang Bo, Feihu Bai

**Affiliations:** a The Third School of Clinical Medicine, Ningxia Medical University, Yinchuan, China; b Department of Gastroenterology, Endoscopic center, People’s Hospital of Ningxia Hui Autonomous Region, Yinchuan, China; c Tongji Hospital, School of Medicine, Tongji University, Shanghai, China; d Institute of Digestive Disease, School of Medicine, Tongji University, Shanghai, China; e Department of Gastroenterology, The Second Affiliated Hospital of Hai Nan Medical College, Haikou, China; f The Gastroenterology Clinical Medical Center of Hainan Province, Haikou, China; g Department of Gastroenterology, The Second Affiliated Hospital of Guangzhou University of Chinese Medicine, Guangzhou, China; h China Center for Helicobacter Pylori Molecular Medicine, Shanghai, China; i Department of Hepatobiliary Surgery, People’s Hospital of Ningxia Hui Autonomous Region, Yinchuan, China

**Keywords:** *Helicobacter pylori*, whole-genome sequencing, antibiotic resistance, genotypic, phenotypic, lineage

## Abstract

Antibiotic resistance is the most important factor leading to failed Helicobacter pylori eradication therapy, and personalized treatment based on antibiotic susceptibility is becoming increasingly important. To strengthen the understanding of antibiotic genotypic resistance of H. pylori and identify new antibiotic resistance loci, in this study, we identified phenotypic resistance information for 60 clinical isolates and compared the concordance of phenotypic and genotypic resistance using whole-genome sequencing (WGS). Clarithromycin and levofloxacin genotypic resistance was in almost perfect concordance with phenotypic resistance, with kappa coefficients of 0.867 and 0.833, respectively. All strains with the R16H/C mutation and truncation in *rdxA* were metronidazole resistant, with 100% specificity. For other genes of concern, at least one phenotypically sensitive strain had a previous mutation related to antibiotic resistance. Moreover, we found that the A1378G mutation of HP0399 and the A149G mutation of *FabH* might contribute to tetracycline resistance and multidrug resistance, respectively. Overall, the inference of resistance to clarithromycin and levofloxacin from genotypic resistance is reliable, and WGS has been very helpful in discovering novel H. pylori resistance loci. In addition, WGS has also enhanced our study of strain lineages, providing new ways to understand resistance information and mechanisms.

## INTRODUCTION

As one of the most prevalent pathogens in the world, Helicobacter pylori has infected more than half of the world’s population in the last decades since its discovery. Studies have shown that infection is strongly associated with chronic gastritis, peptic ulcers, gastric cancer, and mucosa-associated lymphoid tissue (1–3). It is well known that eradicating H. pylori can prevent the progression of associated diseases and reduce the risk of gastric cancer (4). Multiple consensus guidelines recommend that all patients with H. pylori infection should receive eradication therapy, unless there are contraindications (3, 5, 6). In addition, the H. pylori eradication rates have declined significantly due to factors such as antibiotic resistance, with the standard triple therapy eradication rate dropping from >90% to <60% (7). In addition, antibiotic resistance has been further exacerbated by the spread of large-scale empirical eradication therapy and the unregulated use of antibiotics. Antibiotic resistance has become the main cause of H. pylori eradication failure (8, 9). H. pylori strains isolated from patients for whom eradication therapy failed were found to be nearly 100% resistant to metronidazole, and more than 60% were resistant to clarithromycin and levofloxacin (10–12). Hence, the antibiotic resistance of H. pylori is alarming.

Personalized therapy based on antibiotic resistance testing, which includes both phenotypic resistance and genotypic resistance testing, might be a major strategy to overcome eradication failure. Traditional antibiotic susceptibility testing requires the isolation and culture of strains from gastric mucosal specimens. The demanding culture conditions, long culture time, and low culture success rate associated with traditional antibiotic susceptibility testing hamper widespread clinical applications (13). H. pylori genotypic resistance testing might be a good alternative to phenotypic resistance testing. Some genetic mutation loci have been reported to contribute to the H. pylori phenotype, and phenotypic resistance tests for clarithromycin and quinolones have been partially replaced by genotypic resistance tests (14, 15). However, the loci of H. pylori conferring resistance to other antibiotics are controversial (16, 17), and further studies are needed to determine which genetic mutations are responsible for resistance.

Recently, whole-genome sequencing (WGS) has been widely applied to discover new antibiotic resistance loci and even as a partial replacement for traditional antibiotic susceptibility testing (18, 19). Several studies have reported local WGS findings that have contributed to the discovery of new mutant loci for antibiotic resistance genes. However, these results remain inconsistent in terms of phenotypic resistance and genotypic resistance concordance for clarithromycin and quinolone (17). Owing to the small sample sizes in these studies, the mechanisms of resistance to some antibiotics to which H. pylori is rarely resistant (e.g., amoxicillin) remain unclear.

In view of the differences in H. pylori characteristics in different regions, as well as the controversy and potential of WGS to detect antibiotic resistance and the strain spectrum, China still lacks WGS data on clinically isolated H. pylori strains. We performed WGS on 60 clinically isolated H. pylori strains stored in the China Center for H. pylori Molecular Medicine, compared the genotypes and phenotypes of strains resistant to six antibiotics, and detected some novel resistance mutation loci. In addition, we identified the strain lineage using WGS.

## RESULTS

### Clinical data and phenotypic resistance.

We successfully recovered and identified 60 clinical isolates of H. pylori. Demographic data and clinical characteristics were also analyzed. The mean age of the strain hosts was 43.78 ± 12.06 years. Thirty-two samples were isolated from male patients, and the remaining 28 samples were from female patients. Half of the patients had a primary infection with no history of eradication treatment.

**FIG 1:**
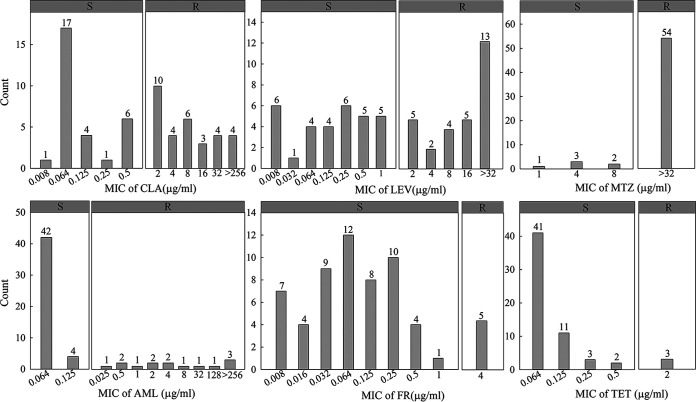
MIC distribution for strains sensitive and resistant to CLA, LEV, AML, TET, FR, and MTZ. Antibiotic resistance in 60 clinical strains of Helicobacter pylori was determined by the Etest. Resistance to CLA, LEV, TET, MTZ, and AML was determined according to the breakpoints published in the European Committee on Antimicrobial Susceptibility Testing (EUCAST) guidelines. The breakpoint of FR resistance was 4 μg/mL according to a previous report. R denotes resistant and S denotes susceptible. MTZ, metronidazole; CLR, clarithromycin; AML, amoxicillin; LEV, levofloxacin; TET, tetracycline; FR, furazolidone.

**FIG 2:**
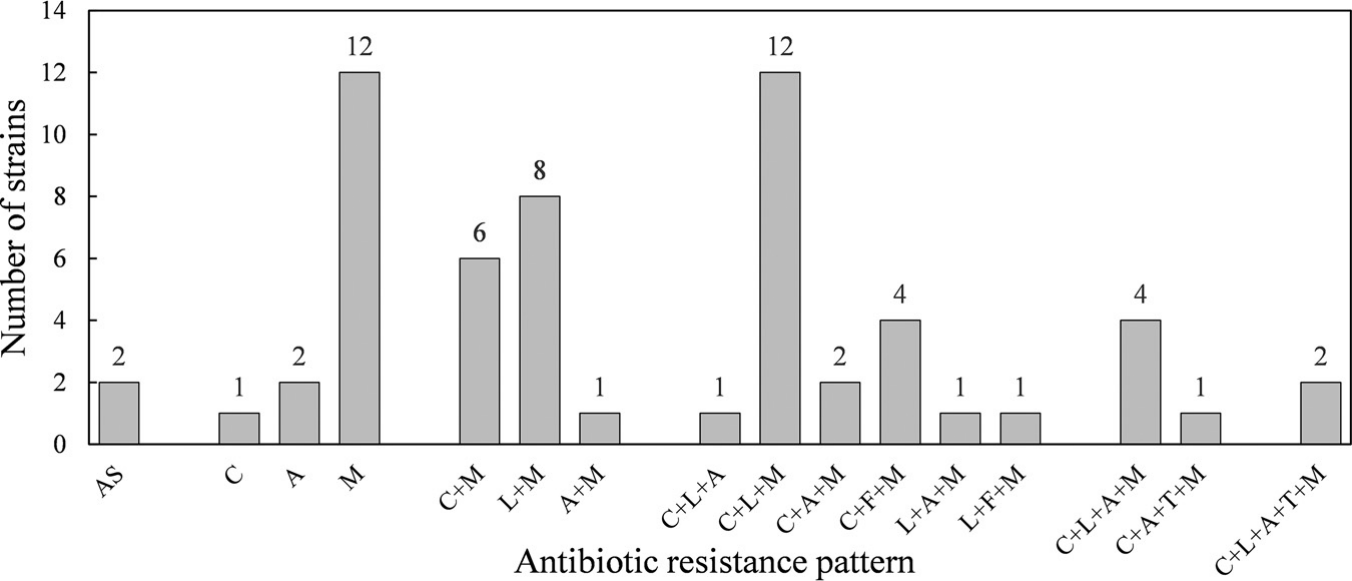
Distribution of antibiotic resistance patterns. Sixty clinical isolates showed different resistance patterns, with all-susceptible, single-antibiotic-resistant, dual-antibiotic-resistant, triple-antibiotic-resistant, tetra-antibiotic-resistant, and penta-antibiotic-resistant strains. AS, sensitivity to all antibiotics; M, MTZ, metronidazole; C, CLR, clarithromycin; A, AML, amoxicillin; L, LEV, levofloxacin; T, TET, tetracycline; F, FR, furazolidone.

The MIC distribution of the six antibiotics is shown in Fig. 1, and the specific MIC values for 60 clinical isolates are presented in Table S1. The total phenotypic antibiotic resistance rates were as follows: metronidazole, 90% (54/60), clarithromycin, 51.67% (31/60), levofloxacin, 48.33% (29/60), amoxicillin, 23.33% (14/60), tetracycline, 5.00% (3/60), and furazolidone, 8.33% (5/60) (Table 1). Two isolates were sensitive to all six antibiotics tested. Sex and age were not significantly associated with antibiotic resistance. The resistance rates of the isolates with a history of eradication with clarithromycin and amoxicillin were significantly higher than those without eradication history (*P = *0.000 and *P = *0.000, respectively), but the rates of resistance to other antibiotics were not related to eradication history. Overall, the secondary resistance rates were higher than the primary resistance rates, except those for metronidazole and furazolidone (Table 1). Multidrug resistance (MDR) accounted for 46.67% of strains, with two strains being simultaneously resistant to five antibiotics, and 26.67% of the strains were simultaneously resistant to clarithromycin, levofloxacin, and metronidazole. The distribution of antibiotic resistance patterns is shown in Fig. 2.

**TABLE 1:** The distribution of antibiotic resistance of H. pylori isolated strains by sex, age, and infection status

		Antibiotic-resistant strain, *n* (%)					
Group	Total, *n* (%)	CLA	LEV	MTZ	AML	TET	FR
Total	60 (100)	31 (51.67)	29 (48.33)	54 (90.00)	14 (23.33)	3 (5.00)	5 (8.33)
Sex							
Female	28 (46.67)	16 (57.14)	13 (46.43)	26 (92.85)	6 (21.43)	1 (3.57)	1 (3.57)
Male	32 (53.33)	15 (46.88)	16 (50.00)	28 (87.5)	8 (25.00)	2 (6.25)	4 (12.5)
*P* value		0.427	0.782	0.675	0.770	0.635	0.212
Age							
18–35	14 (23.33)	6 (42.86)	5 (35.71)	11 (78.57)	3 (21.43)	0 (0.00)	1 (7.14)
35–50	31 (51.67)	16 (51.61)	16 (51.61)	29 (93.55)	9 (29.03)	2 (6.45)	4 (12.90)
50–75	15 (25.00)	9 (60.00)	8 (53.33)	14 (93.33)	2 (13.33)	1 (6.67)	0 (0.00)
*P* value		0.653	0.555	0.291	0.523	1.000	0.413
Eradication history							
None	30 (50.00)	8 (26.67)	12 (40.00)	28 (93.33)	1 (3.33)	0 (0.00)	3 (10)
Yes	30 (50.00)	23 (76.67)	17 (56.67)	26 (86.67)	13 (43.33)	3 (10.00)	2 (6.67)
*P* value		0.000	0.196	0.671	0.000	0.237	1.000
No. of treatments							
One	19 (63.33)	14 (73.68)	11 (57.89)	15 (78.94)	6 (31.58)	0 (0.00)	1 (5.26)
Two	8 (26.67)	6 (76.67)	4 (50.00)	8 (100.00)	5 (62.50)	1 (12.5)	1 (12.5)
Three	2 (6.67)	2 (100.00)	1 (50.00)	2 (100.00)	1 (50.00)	1 (50.00)	0 (0.00)
Five	1 (3.33)	1 (100.00)	1 (100.00)	1 (100.00)	1 (100.00)	1 (100.00)	0 (0.00)

### Mutation analysis using WGS.

**(i) Clarithromycin resistance.** Mutations in the peptidyl transferase region of domain V of *23S rRNA* are associated with clarithromycin resistance (20). In total, 31 strains were phenotypically resistant to clarithromycin, of which 27 had the A2143G mutation (Table S2). Another four strains were phenotypically resistant but genotypically sensitive, and one of them was associated with a high MIC (>256 μg/mL). None of the isolates contained the A2142G mutation. We performed kappa concordance analysis with the A2143G mutation as genotypic resistance versus phenotypic resistance, with a kappa coefficient of 0.867 and a 95% confidence interval (CI) of 0.742 to 0.992 (Table 2). In addition, we examined other mutation loci in *23S rRNA* that might be associated with clarithromycin resistance, such as G2111A, A2115G, A2144G, A2116G, C2694A, and T2717C, which were previously considered to be associated with low levels of resistance, as well as C2173T and G2212A, which occurred only in resistant strains, but none of these mutations were found in our isolates (21–25). Moreover, the T2182C mutation has been reported to be associated with low levels of antibiotic resistance (26), and 83% of our isolates had this mutation, with MIC values ranging from 0.064 to 256 μg/mL. Furthermore, we analyzed the mutations associated with *rpl22* and *infB* (27, 28), which were reported to confer synergistic resistance with *23S rRNA* mutations (Table S2). However, we did not find mutations in *rpl22* that were reported to contribute to clarithromycin resistance. One isolate had both the *23S rRNA* (A2143G) and *infB* (G160A) mutations, with an MIC of >256 μg/mL.

**TABLE 2:** Consistency of phenotypic resistance with genotypic resistance

Antibiotic	Genotypic resistance or susceptibility	No. of phenotypically resistant or susceptible strains		Sensitivity (%)	Specificity (%)	Accuracy (%)	Kappa coefficient (95% CI)	*P* value
		R	S					
CLA	R	27	0	87.10	100.00	93.33	0.867 (0.742–0.992)	0.000
	S	4	29					
LEV	R	26	2	89.66	92.86	91.67	0.833 (0.693–0.973)	0.000
	S	3	29					
MTZ	R	17	0	31.48	100.00	40.00	0.084 (0.012–0.158)	0.170
	S	37	6					
TET	R	2	0	66.67	100.00	98.33	0.792 (0.396–1.188)	0.000
	S	1	57					
AML	R	12	31	85.71	32.61	45.00	0.106 (−0.035–0.248)	0.310
	S	2	15					
FR	R	0	0			93.33		
	S	5	55					

**(ii) Levofloxacin resistance.** Levofloxacin resistance is usually thought to be caused by mutations in the quinolone resistance-determining region of *gyrA* (16). In total, 29 isolates were phenotypically resistant to levofloxacin, 26 of which had *gyrA* (N87I/N87K/N87Y/D91N/D91G) mutations, with no D91Y mutations identified (Table S3). R130K and S63P were previously thought to be associated with high levels of levofloxacin resistance (29). No S63P mutations were detected in our isolates. The R130K mutation was found in only one isolate, which also had the N87K mutation and was associated with high MIC values. Some mutations in *gyrA* suspected to be associated with levofloxacin resistance were incidental in this study and did not correlate with phenotypic resistance (17, 28) (Table S3). In addition, A407T occurred only in phenotypically resistant strains. For *gyrB*, the D481E and R484K mutations, which were previously thought to be associated with levofloxacin resistance (30, 31), occurred only in phenotypically resistant strains (Table S3). In addition, *gyrB* mutations (N573D/S, A584V) occurred only in phenotypically resistant strains. We used the N87K/I/Y and D91N/G mutations of *gyrA* in a kappa concordance analysis of levofloxacin genotypic resistance with phenotypic resistance, finding a kappa coefficient of 0.833 and a 95% CI of 0.693 to 0.973 (Table 2).

**(iii) Metronidazole resistance.** Most current studies suggest that metronidazole resistance is closely related to mutations in and the inactivation of *rdxA*, encoding oxygen-insensitive NADPH nitroreductase, and *frxA*, encoding NADPH flavin oxidoreductase (32, 33), but there is no definitive conclusion. Therefore, we described all genetic mutations associated with metronidazole resistance. The R16H/C and M21A mutations of *rdxA* are considered most strongly associated with metronidazole resistance (34). Unfortunately, we found that only 12 of the 54 phenotypically resistant strains had R16 H/C mutations (Table S4). Our isolates did not harbor an M21A mutation. Moreover, the A68V mutation occurred only in metronidazole-resistant strains. Furthermore, we identified mutations in *rdxA* and *frxA* that were previously confirmed using natural transformation assays and suspected to be associated with metronidazole resistance, which included some studies on clinical isolates, but none had any correlation with metronidazole resistance (Table S4). Interestingly, we found truncated alterations in *rdxA* and *frxA* only in resistant strains (Table S5). The genotypic resistance, defined as *rdxA* truncations and R16H/C mutations, correlated slightly with the metronidazole phenotypic results (kappa coefficient, 0.084; 95% CI, 0.012 to 0.158), but the specificity was 100% (Table 2). In addition, we investigated previously reported genetic mutations associated with metronidazole resistance, including *RclC*, *HP0370*, *HP0918*, *Rpsu*, *Fur*, *RecA*, *Ribf*, and *Omp11* (29, 35–38), and found that only V265I of *HP0370* and A51V/T of *HP0918* occurred at higher frequencies in our samples, and these were harbored only by resistant strains. In addition, we report the following novel findings: N118K in *Fur*, Q242K in *Ribf*, and K219Q/N and H705fs in *Omp11*, which were found only in resistant strains (Table S4).

**FIG 3:**
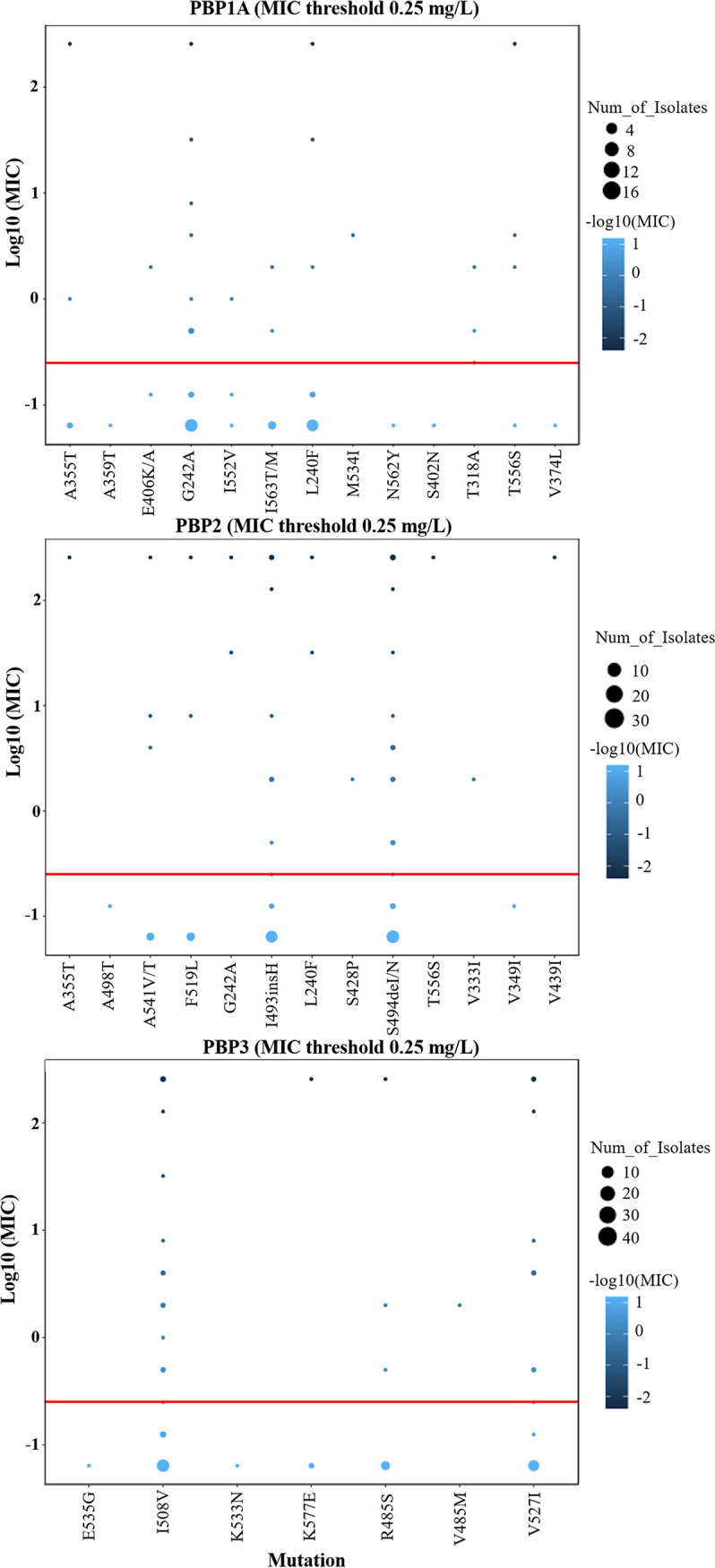
Consistency between amino acid substitutions near SXN/SXXK/KTG of PBP1A, PBP2, and PBP3 motifs and the MIC of amoxicillin. The horizontal axis is the amino acid substitution site, the vertical axis is the value of the logarithm of 10 for the lowest inhibitory concentration, and the red line represents the drug resistance breakpoint.

**FIG 4:**
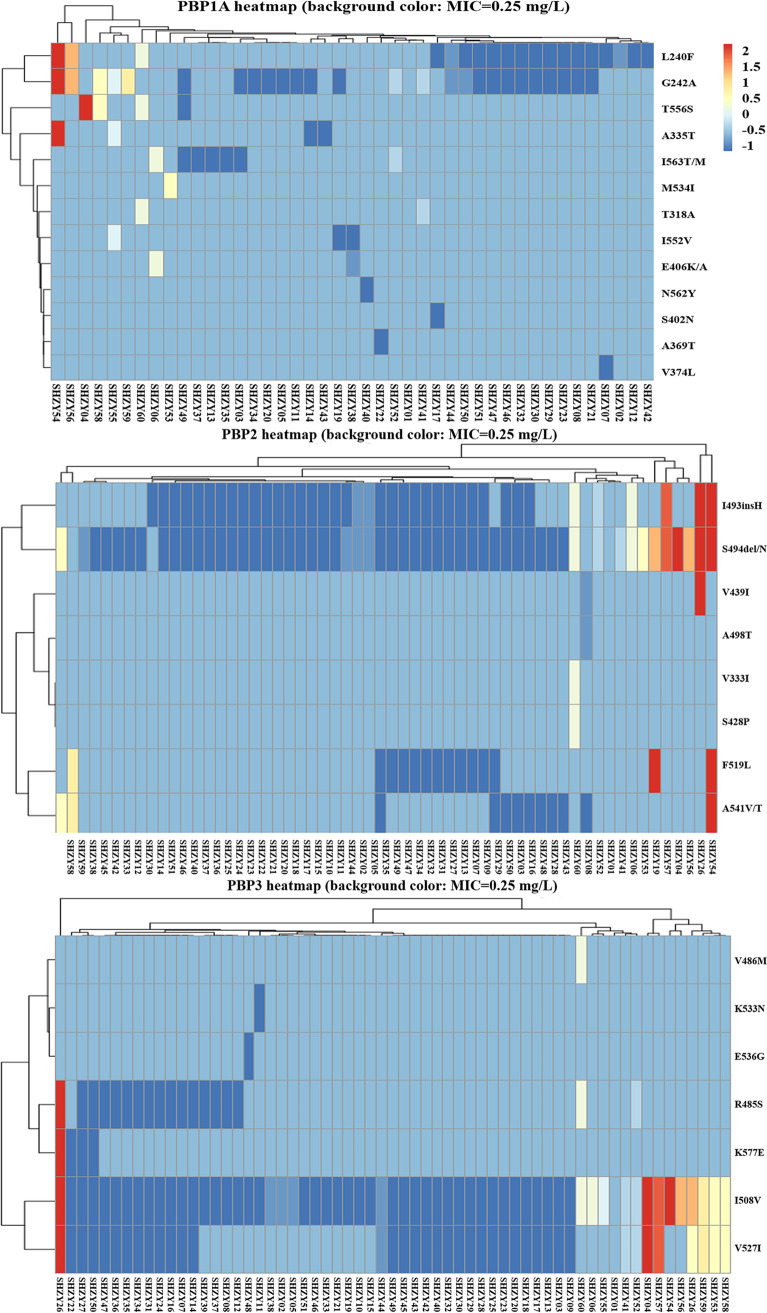
Visualization of MIC and sample clustering of amino acid substitutions near SXN/SXXK/KTG for PBP1A, PBP2, and PBP3 motifs. The MIC values in the graph are the result of taking the logarithm of 10. The light blue background represents an MIC-associated truncation.

**(iv) Amoxicillin resistance.** Mutations in or near the penicillin-binding protein motifs SXXK, SXN, and KTG of the penicillin-binding protein genes *PBP1A*, *PBP2*, and *PBP3* are generally considered associated with amoxicillin resistance (39, 40). We evaluated 60 clinical isolates for variants in genes encoding penicillin-binding proteins, including 14 phenotypically resistant strains. Surprisingly, S402G and N562Y in SXN occurred only in two sensitive strains, whereas T593A/R and S414N/R occurred in both resistant and sensitive strains. T556S in KTG was detected in only one resistant strain (Table S6). These five point mutations were previously validated using natural transformation assays and verified in clinical strains with amoxicillin resistance (14, 41). In addition, we evaluated other possible antibiotic resistance mutation sites in *PBP1A* and partial mutation sites in *PBP2* and *PBP3* that were synergistic with *PBP1A* resistance mutations in previous reports but did not correlate with the amoxicillin phenotypic resistance (31, 42). Unexpectedly, we found that T318A, located near SXXK of the *PBP1A* motif, was associated with amoxicillin resistance (*P = *0.01; Table S6). Furthermore, we found that both sensitive and resistant strains had different degrees of mutations in or near SXN/SXXK/KTG (Table S7). The kappa concordance analysis showed a kappa coefficient of 0.106, with a 95% CI of −0.035 to 0.248 (Table 2). Moreover, we performed bubble map analysis of mutations occurring in and around the SXN/SXXK/KTG motifs of *PBP1A*, *PBP2*, and *PBP3*; however, we did not find a strong association between the amoxicillin resistance phenotype and coexisting mutations (Fig. 3). By performing clustering analysis, we found that simultaneous L240F and G242A mutations in the *PBP1A* gene, I493insH and S494delN mutations in the *PBP2* gene, and I508V and V527I in the *PBP3* gene seemed to be associated with a high MIC and confer resistance (Fig. 4). Meanwhile, the resistant strain SHZY54 associated with a high MIC had mutations near the PBP motifs of *PBP1A*, *PBP2*, and *PBP3*. Furthermore, L378F and D131E in *hefC* and G228W in *hofH* were previously found to be significantly associated with the *in vitro* induction of amoxicillin resistance using WGS (43). However, our results were not consistent with these findings (Table S6).

**(v) Tetracycline resistance.** The main molecular mechanism underlying tetracycline resistance in H. pylori is associated with single, double, or triple base mutations in the *16S rRNA* gene AGA926-928 (44, 45). Three isolates were phenotypically resistant to tetracycline (SHZY01, SHZY41, and SHZY52) in our study. After a sequence comparison, we did not find the AG926-927 mutation, whereas the A928C mutation occurred in the resistant strains SHZY01 and SHZY41. The AGA926-928 mutation in *16S rRNA* was in substantial concordance with the tetracycline phenotype (kappa coefficient, 0.792; 95% CI, 0.396 to 1.188), with a specificity of 100% (Table 2). Meanwhile, A980G and G961A occurred in SHZY01 and SHZY52, respectively (Table S8). In addition, we found that the A1378G mutation in HP0399, encoding 30S ribosomal protein S1, was strongly correlated with tetracycline phenotypic resistance (*P = *0.002; Table S8).

**(vi) Furazolidone resistance.** Five furazolidone-resistant strains were included in our isolates. We chose to examine *porD* (G353A, A356G, C357T) and *oorD* (A41G, A122G, C349A[G]), point mutations previously reported to be associated with furazolidone resistance. However, these mutations were not found in our strains (46). Furthermore, the high frequency of mutant loci in the *porD* and *oorD* genes, described in a previous study reporting unknown furazolidone resistance, was still present in our isolates but did not correlate with furazolidone resistance (47) (Table S8).

**(vii) MDR.** Unexpectedly, Fisher’s test results for all possible variants revealed that the A149G mutation in *FabH* was closely associated with MDR (*P = *0.009; Table S9). In addition, we found that four strains (SHZY05, SHZY06, SHZY23, and SHZY56) were genotypically sensitive and phenotypically resistant to clarithromycin and had an A149G mutation in *FabH* (highlighted in yellow in Table S9).

### Evolutionary tree analysis of H. pylori.

**FIG 5:**
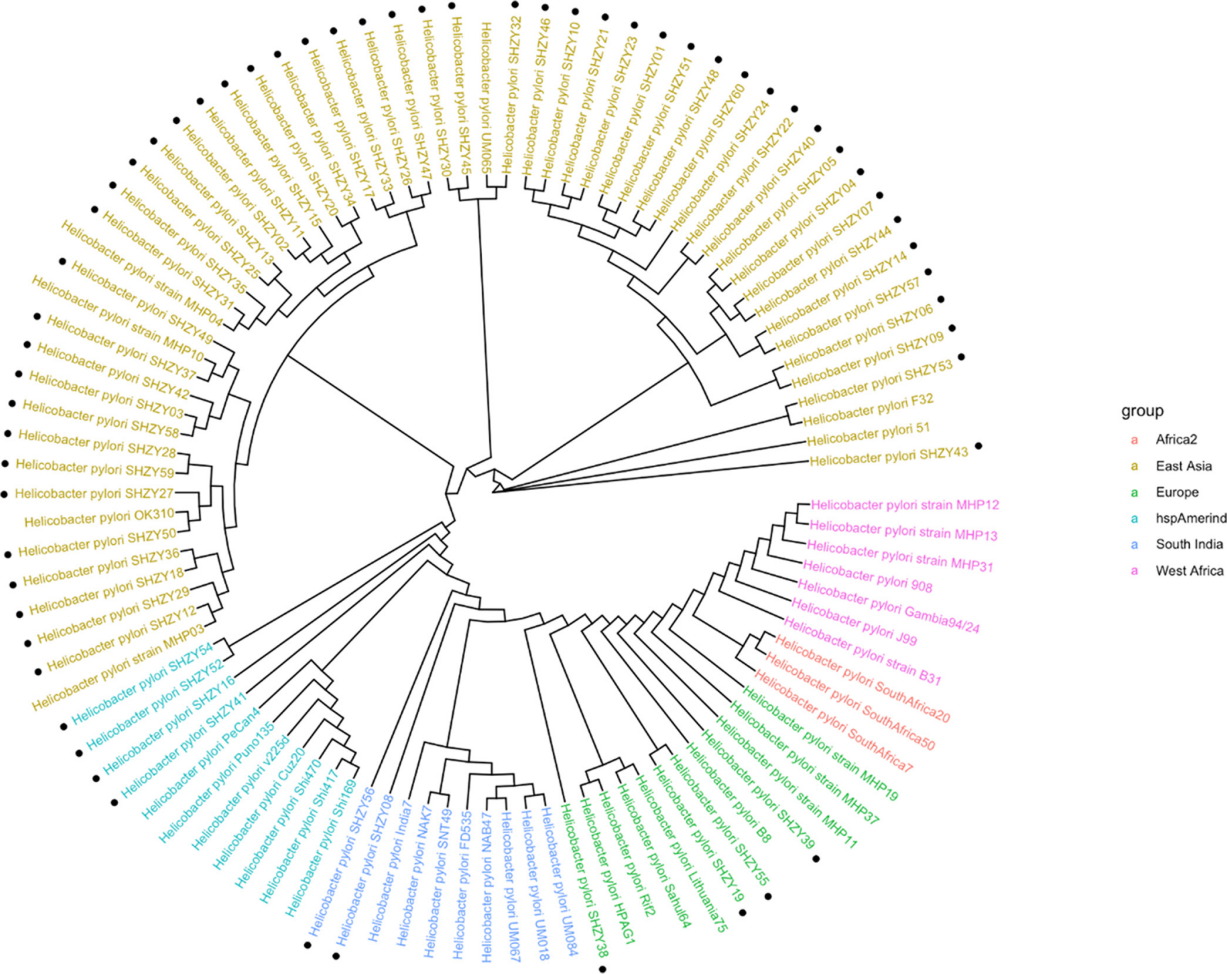
Phylogenetic tree based on the amino acid and nucleotide sequences of 100 genes shared across 100 Helicobacter pylori genomes. Those marked with black dots are the 60 genomes evaluated in our study. The additional 40 genomes were previously described in the literature.

We used WGS data to compare the 60 strains from this study with the 40 complete genomes already classified in the database to construct a phylogenetic tree to determine where the 60 strains clustered. As expected, the majority of our strains (83.33%, 50/60) belonged to the hpEastAsia clade (Fig. 5). Two strains (SHZY08 and SHZY56) clustered with the South India branch, four (SHZY19, SHZY38, SHZY39, and SHZY55) clustered with the European branch, and another four (SHZA16, SHZY41, SHZY52, and SHZY54) clustered with the Amerind branch. None of the isolates clustered with the West Africa or Africa2 branch. The four isolates clustered in Amerind had the same susceptibility or resistance to levofloxacin and clarithromycin, whereas three of them were resistant to amoxicillin.

## DISCUSSION

Declining H. pylori eradication rates have led to a focus on precision treatment (48), which might be able to specify the resistance of H. pylori using genotypic testing to select antibiotics for personalized treatment; however, the results from various studies are inconsistent (17, 34, 49). In this study conducted in China, we identified the phenotypic resistance of 60 clinical isolates of H. pylori to six antibiotics and performed WGS to identify gene mutations associated with this trait and discover new antibiotic resistance sites.

The resistance rate to clarithromycin, which is a key component of nonbismuth quadruple therapy, is rapidly increasing worldwide (50, 51). The point mutations A2143G and A2142G/C in the *23S rRNA* gene have been reported to be associated with clarithromycin-resistant strains of H. pylori isolated from Western countries (52–54). Gong et al. (55) noted that all patients developed clarithromycin resistance after failure of clarithromycin-based triple therapy for eradication, whereas *23S rRNA* sequencing revealed they all had A2143G or A2142G mutations. Our results showed that the samples with the A2143G mutation in *23S rRNA* were all clarithromycin-resistant strains. Hence, the A2143G mutation is highly specific for putative clarithromycin sensitivity and has almost perfect concordance. Similar to some previously reported results, the concordance was high (14, 16, 49). In addition, the reliability of the results was confirmed by previous validation work from this group in a large national multicenter study based on Sanger sequencing (12). Of course, there were also four strains with inconsistent phenotypic and genotypic resistance. One possible explanation for the different phenotypic–genotypic results is the following. Since all four inconsistent isolates were MDR and had the A149G mutation in *FabH*, we hypothesized that they might be associated with MDR. Of note, none of our isolates had the A2142G/C mutation. In addition, we found that the G2864A mutation occurs only in resistant strains, which further complements the clarithromycin resistance locus. Thus, clarithromycin genotypic resistance testing can replace phenotypic resistance testing.

Levofloxacin is crucial in the second-line eradication treatment of H. pylori infection and is recommended as a first-line antibiotic in some countries (56, 57). Almost half of our isolates were resistant to levofloxacin. It is generally believed that levofloxacin resistance is associated with the mutations N87K/I/Y and D91N/Y/G in *gyrA* (16). It has been reported that 97.7% of levofloxacin-resistant strains have the *gyrA* mutation N87 K/I/Y or D91 N/Y/G (58). We investigated the full-length sequence of *gyrA* and found that the mutation rate at amino acid positions 87 and 91 was 89.66% in phenotypically resistant strains. Although there were five isolates with different phenotypic and genotypic results, the kappa coefficient showed almost perfect agreement between the overall phenotypic and genotypic resistance. In fact, not all studies are highly consistent with the study by Domanovich-Asor et al. (17), which showed poor agreement between phenotypic and genotypic resistance to levofloxacin. Overall, most of the levofloxacin resistance can be explained by amino acid mutations at positions 87 and 91 in *gyrA*, but there are other reasons for levofloxacin resistance, such as mutations in *gyrB* and other mutations mentioned in our results.

The rate of resistance to metronidazole, a common antibiotic used in triple therapy, has reached 100% in some areas, and its resistance loci remain unclear (59). *rdxA* and *frxA* mutations are thought to be highly correlated with phenotypic resistance to metronidazole. The R16H/C mutation in *rdxA* and truncation of *rdxA* and *frxA* occurred in 35% of the isolates in our study, and all of them were phenotypically resistant strains. Chu et al. verified via a natural transformation experiment (15) and WGS conducted in New York that the occurrence of R16H mutations or truncations in *rdxA* is highly correlated with phenotypic resistance, with a kappa coefficient of 0.76 (49). This suggests that the R16H/C mutation and truncation of *rdxA* are useful in determining metronidazole resistance with 100% specificity. However, there were 37 phenotypically resistant strains without the R16H/C mutation or truncation in our study. Another two studies showed that owing to the polymorphism of the variants, it was not possible to infer a resistance phenotype for metronidazole based on the occurrence of distinct single nucleotide polymorphisms in *frxA* and *rdxA* (16, 17). The concordance between phenotypic and genotypic resistance was low. Furthermore, we investigated other reported gene mutations that might be associated with metronidazole resistance, such as mutations in *frxA*, *rpsu*, *RclC*, *HP0918*, *HP0370*, and other genes, and no meaningful results were found. This means that the inactivation of *rdxA* function is responsible for the development of resistance to metronidazole in some strains. Furthermore, there are many other mechanisms of metronidazole resistance, including altered drug uptake, efflux from the efflux pump system, and permeability of biofilms, which need to be considered (32, 60). In general, there are still some limitations in determining phenotypic resistance to metronidazole alone.

Amoxicillin is a crucial antibiotic used in bismuth quadruple therapy, concomitant therapy, and high-dose diphtheria regimens for H. pylori eradication (6). The amoxicillin resistance rate is increasing annually (61). In total, 14 isolates in our study were resistant to amoxicillin. We investigated the mutations in the PBP motifs SXXK, SXN, and KTG of *PBP1A*, *PBP2*, and *PBP3* using WGS. Both sensitive and resistant strains had different degrees of mutations in and near the motifs SXXK, SXN, and KTG of PBP (Table S7). Only one resistant strain had an amino acid substitution in the *PBP1A* gene at the site previously reported to confer amoxicillin resistance (S414N/R, T556S) (14, 41). Rimbara et al. suggested that *PBP1A*, *PBP2*, and *PBP3* mutations have synergistic effects on amoxicillin resistance (62). Our clustering results seem to confirm this, with the highly resistant strain SHZY54 mutated near the motifs SXXK, SXN, and KTG of *PBP1A*, *PBP2*, and *PBP3*. However, for mutations in and around the *PBP1A*, *PBP2*, and *PBP3* motifs, bubble plots showed no clear association between the amoxicillin genotypic and phenotypic results (Fig. 3). Kappa concordance analysis yielded the same results (Table 2). In addition, other reported genetic mutations associated with amoxicillin resistance were not observed in our study (Table S6). This suggests that there is large heterogeneity in amoxicillin resistance and that the resistance mechanisms of different isolates cannot be explained by a single amino acid substitution in PBP motifs (17). We also need to consider the isolation status of the strain, host differences, degree of coccoid transformation, and length of survival time.

The rate of resistance to tetracycline is low worldwide but is still increasing (63). Only three tetracycline-resistant strains were detected among our isolates, and we detected a mutation in the *16S rRNA* gene, which was previously considered to be related to tetracycline resistance, using WGS (16, 58). We found only two strains with the A928C mutation, and both were tetracycline-resistant strains, which means that the specificity of tetracycline resistance based on the AGA926-928TTC mutation in *16S rRNA* reached 100%. In addition, we found that the A1378G mutation in *HP0399* occurs only in tetracycline-resistant strains. On the one hand, tetracycline binds to the 30S subunit of the bacterial ribosome and inhibits protein synthesis outside (64). On the other hand, tetracycline also inhibits the binding of nucleoprotein bodies to released factors (65, 66), preventing the release of synthesized peptide chains and thus inhibiting protein synthesis. *HP0399* encodes the 30S ribosomal protein S1, and mutations in this gene weaken the binding of nucleoprotein to released factors, allowing the synthesized peptide chains to be normally released and the bacteria to continue to grow and multiply. This might be another mechanism of resistance to tetracycline, which requires further study.

Furazolidone is widely used as an antibiotic in China for the eradication of H. pylori. Its efficacy and safety have also been well demonstrated (67–70). There were five furazolidone-resistant strains among our isolates, and the previously described mutations in the *porD* and *oorD* genes associated with furazolidone resistance were not found (46). In the future, as the use of furazolidone increases, an increasing number of resistant strains might appear in clinical practice, and the resistance mechanisms need to be further studied.

Notably, MDR was common, and nearly half of the isolates exhibited this MDR in our study. The A149G mutation in *FabH* was strongly correlated with MDR (*P = *0.009). As an important constituent of cell membranes, alterations in lipid metabolism have an effect on antibiotic resistance, and previous studies have shown that the type II fatty acid synthesis pathway in bacteria is associated with MDR (71–73) and that *FabH* plays a key role in the overall bacterial fatty acid synthesis pathway (74). *FabH* is commonly found in many clinical pathogens, such as Gram-positive bacteria, Gram-negative bacteria, chlamydia, anaerobic bacteria, *Mycobacterium*, and many protozoa, and its gene sequence is highly conserved in three-dimensional structures. Structural analysis identified *HP0202* as being active in the H. pylori 26695 genome with a homologue of *FabH*. *FabH* has been shown to be involved in fatty acid anabolism in H. pylori (75). The role of *FabH* gene mutations in H. pylori MDR is unclear. In the future, we might need to explore the mechanism of *FabH*-mediated MDR in H. pylori, which is an important next step.

H. pylori is divided into seven different lineages according to geographical region (76). Phylogenetic analysis revealed that the 60 isolates from one region in this study did not have consistent H. pylori lineages. In addition to the majority of the strains belonging to HpEastAsia, there were scattered distributions among HspAmerind, HpEurope, and South India. A modeling study suggests that H. pylori appears to have spread from East Africa approximately 58,000 years ago, with people becoming infected with H. pylori before migrating from Africa (77), and that H. pylori has maintained close contact with human hosts over time. However, the currently existing H. pylori lineage has been at the intersection of humans that coevolved with the HpAsia2 lineage of H. pylori on the Indian subcontinent and humans from Central Asia with ancestral links to Europe (78). Because of the plasticity of the H. pylori genome, it is expected that these coexisting lineages will converge over time, and thus maintaining the independence of H. pylori in a stable ethnic group seems somewhat difficult. This could be one of the reasons for the distribution of isolates across different lineages. In addition, the increased globalization process might be another major reason for the diversity of our strains. Moreover, we observed that the four isolates that clustered in the Amerind lineage had the same susceptibility or resistance to levofloxacin and clarithromycin, and there was a larger proportion of amoxicillin-resistant strains (3/4, 75%). Considering our small sample size, further studies with more samples are required to determine if there is an association between H. pylori strain lineage and antibiotic resistance.

In this study, we investigated the loci of H. pylori conferring resistance to different antibiotics using WGS. Our results showed almost perfect concordance between genotypic and phenotypic resistance to clarithromycin and levofloxacin, substantial concordance for tetracycline, and slight concordance for the others. However, the R16H/C mutation and truncation in *rdxA* were very specific for determining metronidazole resistance. Therefore, *23S rRNA* and *gyrA* phenotypic testing for clarithromycin and levofloxacin resistance can be implemented to a greater extent to facilitate the implementation of precision therapy. However, it is still challenging to infer resistance results directly from the genotypic profiles of other antibiotics. Of course, the use of WGS technology has also enabled the identification of novel mutations that could be associated with antibiotic resistance, which will help us to further investigate the genotypic resistance mechanisms of H. pylori. At the same time, WGS also allows us to perform a deeper study of the strain lineage, providing a new way to understand antibiotic resistance information and mechanisms. Our study had some limitations. For example, there were fewer tetracycline- and furazolidone-resistant strains in our sample set. In addition, it would be better if we had known the basic characteristics of the sample hosts and previous eradication treatment antibiotic regimens, which would facilitate a clarification of genotypic resistance mechanisms.

## MATERIALS AND METHODS

### Recovery and identification of H. pylori.

We recovered 60 clinical isolates stored at −80°C in the strain bank of the China Center for H. pylori Molecular Medicine. These strains were previously tested by the Kirby-Bauer disk diffusion method and had basic drug sensitivity results. Briefly, the strains were rapidly thawed and recovered within 1 min in a 37°C water bath. Then, 200 μL of the cell suspension was inoculated onto Karmali agar plates supplemented with H. pylori selective supplement (Oxoid, UK) and incubated at 37°C in a microaerobic atmosphere (5% O_2_, 10% CO_2_, and 85% N_2_) for 2 to 3 days. The successful recovery of H. pylori was determined by observing the colony morphology and Gram-stained bacterial morphology and performing urease, peroxidase, and oxidase tests. The need for informed consent was waived owing to the patients being lost to follow-up. We had basic information such as age, sex, eradication history, and region. Not enough clinical information was collected. The protocol for this study was approved by the Ethics Committee of the Outdo China Center for H. pylori Molecular Medicine (YB M-05-01).

### Antibiotic susceptibility testing.

An Etest (BIO-KONT, Wenzhou, China) was used to detect the antibiotic resistance of H. pylori. Pure cultures of H. pylori from the plates were inoculated into 1 mL of sterile saline with a turbidity comparable to McFarland’s turbidity standard number 3. Using a disposable sterile swab, the bacterial solution was inoculated onto MH blood agar plates. The plate was allowed to dry and a single test strip was placed on it. Petri dishes were incubated at 37°C under slightly aerobic conditions for 72 h, and then the MIC was determined. Resistance to clarithromycin, levofloxacin, tetracycline, metronidazole, and amoxicillin was determined according to the breakpoints published in the European Committee on Antimicrobial Susceptibility Testing (EUCAST) guidelines (79). The breakpoint of furazolidone resistance was 4 μg/mL according to a previous report (80). The MIC was measured at the point of complete growth inhibition. For the antibiotic susceptibility assay, the standard strain 26695 was used as a quality control.

### DNA extraction, library preparation, and WGS of H. pylori isolates.

H. pylori clinical isolates were subcultured on blood agar plates for genomic DNA isolation. Three plates were used for each isolate after 3 to 4 days of incubation under microaerophilic conditions at 37°C. The colonies were scraped with a ring and resuspended in saline. Genomic DNA was prepared using the HiPure bacterial DNA kit (Magen Inc.), following the manufacturer’s instructions. DNA quality was assessed using a NanoDrop One spectrophotometer (Thermo Fisher Scientific, Inc.). The genomic library was prepared using the KAPA HyperPlus kit (Illumina, 96, rxns) according to the manufacturer’s instructions. Sequencing was performed on an Illumina MiSeq platform (Illumina, Inc.). The quality of the read files was evaluated using FastQC (v0.11.7) software. Reads and adapters were trimmed using fastp (v0.12.5) software, and the reads were assembled using SPAdes version 3.13.0 software. The number of contigs obtained after assembly ranged from 16 to 172, and the sequence coverage of all isolates was evaluated using the Qualimap (v2.2.1) reporting program, using H. pylori 26695 as a reference. The coverage ranged from 74 to 470 times, with an average depth of 257 times. Quality metric data for all sequenced genomes, including coverage, genome length, and N_50_ and L_50_ values, are presented in Table S10.

### Identification of antibiotic resistance mutations.

We investigated the sequences of *rdxA*, *frxA*, *rpsu*, *RclC*, *HP0918*, *HP0370*, *Ribf*, *RecA*, *Fur*, *Omp11* (associated with metronidazole resistance), *23S rRNA*, *rpl22*, *infB* (associated with clarithromycin resistance), *gryA*, *gryB* (associated with levofloxacin resistance), *PBP1A*, *PBP2*, *PBP3*, *hefC*, *hofH* (associated with amoxicillin resistance), *16s RNA* (associated with tetracycline resistance), *oorD*, and *porD* (associated with furazolidone resistance) in the WGS data. Using 26695 (NC_000915.1) as the reference genome, multiple sequence comparisons were performed to determine the presence of the genes and mutations (31). The correlation between phenotypic and genotypic antibiotic resistance was analyzed. We also analyzed possible novel mutations in phenotypically resistant strains. In addition, MDR was defined as resistance to ≥3 antibiotics of different classes (81), which has raised concerns worldwide. Therefore, we also evaluated the mutations associated with MDR.

### Construction of a phylogenetic tree.

Based on the approach used by Saranathan et al. (49), as described in the literature, we used the PATRIC (version 3.6.10) online tool to construct a phylogenetic tree. We chose the default Codon Trees, which uses the amino acid and nucleotide sequences from a defined number of PATRIC global protein families (82). Both the protein (amino acid) and gene (nucleotide) sequences were used for each of the selected genes from the PGFams. A concatenated alignment of all proteins and nucleotides was written to a PHYLIP-formatted file, and then a partition file for RaxML (83) was generated, describing the alignment in terms of the proteins and then the first, second, and third codon positions. Support values were generated using 100 rounds of the “Rapid” bootstrapping option of RaxML (84). We set the parameters to sequences from 100 genes (38,562 amino acids and 115,686 nucleotides) for phylogenetic tree construction, which included 100 genomes (Table S11 and S12). These genomes included the 60 genomes sequenced in this study and an additional 40 genomes previously reported in the literature (49, 85).

### Statistical analyses.

The phenotypic and genotypic concordance of the antibiotics was quantified using the kappa coefficient. Kappa coefficient values of <0.20, 0.21 to 0.40, 0.41 to 0.60, 0.61 to 0.80, and >0.80 indicated slight, fair, moderate, substantial, and almost perfect concordance, respectively. Qualitative variables were compared using the chi-square test or Fisher’s exact test. For all statistical tests, *P* values of <0.05 were considered statistically significant. All statistical analyses and graph construction were performed using R software (version 3.6.4).

### Data availability.

All 60 sequenced genomes of the H. pylori strains isolated from the 60 clinical specimens investigated in this study were assembled, annotated, and submitted to the NCBI BioProject database under BioProject accession number PRJNA745492. Visit the website at https://www.ncbi.nlm.nih.gov/bioproject/PRJNA745492.
