# Supplementary material for: A Survey of Helicobacter pylori Antibiotic-Resistant Genotypes and Strain Lineages by Whole-Genome Sequencing in China
Source: Antimicrob Agents Chemother. 2022 Jun 2;66(6):e02188-21. doi: 10.1128/aac.02188-21 (PMC9211431; doi:10.1128/aac.02188-21)
Supplement: Supplemental file 1 — Tables S1 to S12. Download aac.02188-21-s0001.pdf, PDF file, 0.75 MB [file aac.02188-21-s0001.pdf]

Table S1 MIC values of 60 clinical isolates

Sample	Gender	Age(years)	Eradication history	MIC(μg/ml)					
				Clarithromycin	Levofloxacin	Amoxicillin	Furazolidone	Tetracycline	Metronidazole
SHZY01	female	44	3	32	0.125	0.25	0.125	2	> 32
SHZY02	Male	30	0	0.008	4	0.125	0.25	0.064	> 32
SHZY03	Male	28	0	0.5	0.25	0.064	0.125	0.125	> 32
SHZY04	female	72	1	8	8	256	0.032	0.064	> 32
SHZY05	Male	47	0	2	16	0.125	0.5	0.064	> 32
SHZY06	female	28	2	> 256	0.5	2	0.5	0.25	> 32
SHZY07	Male	24	0	2	16	0.064	0.008	0.064	> 32
SHZY08	female	47	1	0.125	> 32	0.064	0.008	0.064	> 32
SHZY09	female	38	0	0.064	0.25	0.064	0.032	0.064	> 32
SHZY10	Male	52	0	0.064	0.125	0.064	0.032	0.064	> 32
SHZY11	Male	56	0	2	0.008	0.064	0.25	0.064	> 32
SHZY12	Male	38	0	0.064	2	0.064	4	0.064	> 32
SHZY13	Male	55	2	2	> 32	0.064	0.008	0.064	> 32
SHZY14	Male	34	0	> 256	0.008	0.064	4	0.008	> 32
SHZY15	Male	61	1	> 256	> 32	0.064	0.008	0.064	> 32
SHZY16	female	57	0	0.064	0.008	0.064	0.008	0.064	> 32
SHZY17	Male	42	1	8	0.5	0.064	4	0.125	> 32
SHZY18	female	37	0	2	8	0.064	0.016	0.125	> 32
SHZY19	Male	53	0	0.5	> 32	0.064	0.125	0.064	> 32
SHZY20	female	37	0	0.064	0.064	0.064	0.25	0.064	> 32
SHZY21	female	37	1	4	0.5	0.064	0.008	0.25	> 32
SHZY22	Male	49	0	32	1	0.064	4	0.064	> 32
SHZY23	Male	52	2	2	> 32	0.064	0.25	0.125	> 32
SHZY24	female	42	2	> 256	0.25	0.064	4	0.064	> 32
SHZY25	Male	32	0	0.064	2	0.064	0.032	0.064	> 32
SHZY26	female	45	0	0.064	> 32	> 256	1	0.125	> 32

SHZY27	female	58	0	0.064	4	0.064	0.016	0.064	> 32
SHZY28	female	27	1	8	0.25	0.064	0.032	0.064	4
SHZY29	female	53	1	4	16	0.064	0.064	0.064	> 32
SHZY30	Male	46	0	16	0.5	0.064	0.25	0.064	> 32
SHZY31	Male	46	0	32	0.032	0.064	0.25	0.064	> 32
SHZY32	female	75	0	0.5	0.25	0.064	0.064	0.064	1
SHZY33	Male	39	0	0.064	0.008	0.064	0.016	0.064	> 32
SHZY34	female	75	1	8	0.008	0.064	0.064	0.064	> 32
SHZY35	female	41	0	0.125	16	0.064	0.25	0.064	> 32
SHZY36	female	53	1	0.25	1	0.064	0.125	0.064	> 32
SHZY37	Male	41	0	0.064	0.125	0.064	0.125	0.064	> 32
SHZY38	Male	30	0	0.064	> 32	0.125	0.064	0.008	> 32
SHZY39	Male	48	0	0.064	16	0.064	0.064	0.008	> 32
SHZY40	Male	48	0	0.064	0.008	0.064	0.008	0.064	> 32
SHZY41	Male	63	5	2	8	0.5	0.064	2	> 32
SHZY42	female	32	0	0.5	0.064	0.064	0.032	0.064	> 32
SHZY43	female	46	1	16	> 32	0.064	0.016	0.064	> 32
SHZY44	female	45	1	2	> 32	0.125	0.25	0.25	> 32
SHZY45	female	24	0	0.064	0.064	0.064	0.032	0.064	> 32
SHZY46	female	36	3	2	> 32	0.064	0.125	0.125	> 32
SHZY47	female	36	1	8	> 32	0.064	0.032	0.125	> 32
SHZY48	female	33	1	8	> 32	0.064	0.25	0.5	> 32
SHZY49	female	58	1	16	0.064	0.064	0.25	0.064	> 32
SHZY50	Male	46	0	0.064	0.25	0.064	0.064	0.064	> 32
SHZY51	Male	25	0	0.064	0.5	0.064	0.5	0.125	4
SHZY52	Male	49	2	2	2	0.5	0.032	2	> 32
SHZY53	Male	45	1	4	8	4	0.064	0.064	8
SHZY54	Male	40	1	0.5	1	> 256	0.064	0.125	4
SHZY55	Male	49	2	0.125	2	1	0.064	0.064	> 32
SHZY56	Male	32	2	4	1	32	0.125	0.125	> 32

SHZY57	Male	46	1	0.125	2	128	0.064	0.064	> 32
SHZY58	female	45	2	0.064	0.125	4	0.5	0.064	> 32
SHZY59	Male	24	1	0.5	1	8	0.064	0.125	8
SHZY60	female	36	1	32	> 32	2	0.125	0.5	> 32

Table S2. Mutations related to clarithromycin phenotypic resistance were previously described and first observed in this study

Gene	Mutation	Phenotypic CLA-R		Phenotypic CLA-S		P	Reference	Note
		MR	WR	MS	WS			
23S rRNA	A2143G	27	4	0	29	0.000	(1)	Mutations of the domain V. Missense mutations at well-defined functional codons based on experimental studies. Only A2143G was detected in this study.
	A2142G\C	Not detected						
	G2111A	Not detected					(2)	It is associated with low level MIC resistance
	A2115G						(3) (4)	
	A2144G							
	A2116G						(5)	
	C2694A						(6)	
	T2717C							
	T2182C	29	2	21	8	0.039	(7)	It is still controversial that it is associated with low level of drug resistance
	G1939A	Not detected					(8)	Suspected to be related to clarithromycin resistance, some national strains tested, some not detected
	C2147G						(9)	
	G2172T						(10)	
	T2215C						(11)	
	C2244T						(12)	
	C2173T						(13)	Mutations that occur only in resistant strains based on whole genome sequencing
	G2212A							
rpl22(hp1314)	295 insertion TTCCATGTA	Not detected					(14) (15)	The rpl22 mutations have a synergistic effect with 23S rRNA, resulting in higher MICs.
	226 deletion							

	GTG							No insertional or deletion mutation of rpl22 was detected in this study.
infB (hp1048)	G160A	1	30	1	28	1	(14)	infB mutations have a synergistic effect with 23S rr. SHZY24 isolate had both 23S and infB mutations, but the MIC was 2µg/ml.

Table S3. Mutations related to Levofloxacin phenotypic resistance were previously described and first observed in this study

Gene	Mutation	Phenotypic LEV-R		Phenotypic LEV-S		P	Reference	Note
		MR	WR	MS	WS			
gyrA	N87K	21	8	2	29	0.000	(16, 17)	Associated with high levels of resistance to levofloxacin
	N87I	4	25	0	31	0.049		
	N87Y	1	28	0	31	0.483		
	D91Y	Not detected					(18)	Associated with low levels of resistance to levofloxacin
	D91G	1	28	0	31	0.483		
	D91N	3	26	1	30	0.345		
	A97V	1	28	0	31	0.483	(13)	May be associated with antibiotic resistance
	A88V	1	28	0	31	0.483	(15)	
	R140K	Not detected						
	T239M	1	28	0	31	0.483		
	P188S	Not detected						
	D99V	Not detected						
	V172I	1	28	0	31	0.483		
	R130K	1	28	0	31	0.483	(15)	May be associated with high levels of MIC
	S63P	Not detected						
gyrB	D481E	5	24	0	31	0.022	(19, 20)	May be associated with levofloxacin resistance
	R484K	4	25	0	31	0.048		
	N573D/S	3	26	0	31	0.107	This study	Only in resistant strains
	A584V	3	26	0	31	0.107		

Table S4. Mutations related to metronidazole phenotypic resistance were previously described and first observed in this study

Gene	Mutation	Phenotypic MTZ-R		Phenotypic MTZ-S		P	Reference	Note
		MR	WR	MS	WS			
rdxA	R16H	7	47	0	6	1	(21-23)	Mutations were strongly associated with metronidazole resistant phenotypes
	R16C	5	49	0	6	1		
	M21A	Not detected					(24)	A whole-genome sequencing from Shenzhen, China, showed that only R16H/C and M21A are significant contributors to metronidazole resistance
	G3A	Not detected					(25)	Next-Generation Sequencing results do natural transformation to verify correlation with metronidazole resistance
	C46T							
	G238A							
	G352A							
	C159T	1	53	0	6	1	(26)	Mutations at well-defined functional codons based on experimental studies
	V192fs	1	53	0	6	1		
	V32A	Not detected					(27)	Suspected to be related to metronidazole resistance
	G27C							
	A22S							
	H97T							
	M56V							
	S111L							
	L71P							
	Q113 stop codon							
	R56 stop codon							
	E6 stop codon							
	T31K	52	2	5	1	0.275	(28, 29)	Mutations found in clinical isolates
	D59N	54	0	6	0	1		
	P106L/S	3	51	0	6	1		

	S108A	2	52	1	5	0.275		
	A118S	3	51	1	5	0.351		
	R131K	53	1	6	0	1		
	A68V	6	48	0	6	1	This study	Only in resistant strains
frxA (HP0642)	A70fs	2	52	0	6	1	(13)	Mutations may be associated with metronidazole resistance
	A138T	2	52	0	6	1		
	A32fs	2	52	0	6	1		
	A152V	3	51	0	6	1		
	A153	3	51	0	6	1		
	N111	38	16	6	0	0.179	(30)	
	C193S	46	8	5	1	1		
	-571TA	Not detected					(25)	Next-Generation Sequencing results do natural transformation to verify correlation with metronidazole resistance
RclC （HP0565）	N85D	49	5	6	0	1	(31)	These three novel candidate genes were derived from whole genome sequencing of clinical isolates
HP0370	V265M	11	43	0	6	0.581		These two mutations also have the highest mutation frequency and the strongest correlation in our data.
HP0918	A51V/T	9	45	0	6	1		
Rpsu(HP0562)	G37T	Not detected					(25)	Mutation from clinical isolates
Fur(HP1027)	P114H/S	4	50	0	6	1	(32)	Mutant Fur suppresses SodB expression, which is associated with the development of Metz resistance
	C78Y	Not detected						
	N118K	6	48	0	6	1	This study	Only in resistant strains, may be associated with metronidazole resistance
	H103Y	2	52	1	5	0.275	(33)	Validation of the mutation

RecA(HP0153)	D121fs	2	52	1	5	0.275		associated with metronidazole resistance by constructing a phage library transformation
Ribf(HP1087)	T222A	1	53	0	6	1	(34)	Comparative genome sequencing method demonstrates that mutations in ribf are involved in different stages of metronidazole resistance
	Q242K	15	39	0	6	0.321	This study	Only in resistant strains
Omp11(HP0472)	A1293D	1	53	0	6	1	(34)	Omp11 may be involved in metronidazole resistance by affecting the permeability of cell membranes
	K219Q/N	18	36	0	6	0.165	This study	Only in resistant strains
	H705fs	18	36	0	6	0.165		

Table S5. Truncations identified in rdxA and frxA

Gene	truncations	Strain	MIC(μg/ml)
rdxA	T211stop	SHZY29	> 32
	E133stop	SHZY48	
	Q130 stop	SHZY57	
	Q119 stop	SHZY60	
	E93 stop	SHZY30	
	E75 stop	SHZY03	
	Q50 stop	SHZY52	
frxA	W30	SHZY13	
	W68	SHZY57/SHZY03	
	Q141	SHZY45	

Table S6. Mutations related to amoxicillin phenotypic resistance were previously described and first observed in this study

Gene	Mutation	Phenotypic AML-R		Phenotypic AML-S		P	Reference	Note
		MR	WR	MS	WS			
PBP1A	S402N	0	14	1	45	1	(35, 36)	Already verified in natural transformation experiments； Correlation of mutations with amoxicillin resistance verified in clinical isolates
	S414N/R	1	13	1	45	0.415		
	T556S	1	13	0	46	0.23		
	N562Y	0	14	1	45	1		
	T593A/R	1	13	5	41	1		
	F366L	Not detected					(28, 37)	Mutations may be associated with amoxicillin resistance
	S405N	Not detected						
	A474T	0	14	4	42	0.56		
	T558S	Not detected						
	V45I	3	11	9	37	1		
	V374L	0	14	1	45	1		
	V469M	1	13	1	45	1		
	N504D	13	1	42	4	1		
	S543R	0	14	1	45	1		
	T318A	3	11	0	46	0.01	This study	
PBP2	A296V	Not detected					(38)	Synergistic resistance of PBP1A, PBP2, PBP3
	A541V/T	3	11	7	39	0.685		
	E572G	Not detected						
PBP3	A499V	Not detected						
	E536G	0	14	1	45	1		
hefC	L378F	Not detected					(39)	These two mutations were detected by whole-genome sequencing of five amoxicillin-resistant strains induced in vitro
	D131E							
hofH	G228W							

Table S7. Identification of mutations in SXN/SXXK/KTG and nearby motifs s of PBP1A, PBP2 and PBP3

Strain	MIC(ug/ml)	PBP1A															PBP2								PBP3						
		240	242	318	355	369	374	402	406	414	417	534	552	556	562	563	333	428	439	493	494	498	519	541	485	486	508	527	533	536	577
		L	G	T	A	A	V	S	E	S	S	M	I	T	N	I	V	S	V	I	S	A	F	A	R	V	I	V	K	E	K
SHZY04	256													S							del						V	I			
SHZY26	256																		I	insH	N				S		V	I			E
SHZY54	256	F	A		T															insH	N		L	V			V				
SHZY57	128																			insH	N						V				
SHZY56	32	F	A																		del						V	I			
SHZY59	8		A																	insH	N		L	V			V	I			
SHZY53	4											I									del						V	I			
SHZY58	4		A											S							del			V			V	I			
SHZY06	2								K							T				insH	N						V				
SHZY60	2	F		A										S			I	P		insH	N				S	M	V	I			
SHZY55	1		A		T						T		V														V				
SHZY41	0.5		A	A						N											del						V	I			
SHZY52	0.5		A													M				insH	N				S		V	I			
SHZY01	0.25			A																insH	N						V	I			
SHZY02	0.125	F																	I	insH	N	T					V				
SHZY05	0.125		A																	insH	N						V				
SHZY38	0.125								A				V								del						V				
SHZY44	0.125	F	A																								V	I			
SHZY03	0.064		A													M				insH	N			V			V	I			
SHZY07	0.064	F					L													insH	N		L		S		V	I			
SHZY08	0.064	F	A																					V	S		V				
SHZY09	0.064																			insH	N		L				V	I			
SHZY10	0.064																			insH	N						V				
SHZY11	0.064		A																	insH	N						V		N		
SHZY12	0.064	F																			del				S		V				
SHZY13	0.064															M				insH	N		L				V	I			
SHZY14	0.064		A		T															insH					S		V	I			
SHZY15	0.064																			insH	N						V				
SHZY16	0.064																			insH	N			V	S		V	I			
SHZY17	0.064	F						N												insH	N						V	I			
SHZY18	0.064																			insH	N						V	I			
SHZY19	0.064		A								T		V														V				
SHZY20	0.064		A																	insH	N						V	I			
SHZY21	0.064	F	A																	insH	N						V				
SHZY22	0.064					T														insH	N						V	I			E
SHZY23	0.064	F	A																	insH	N						V	I			
SHZY24	0.064																			insH	N				S		V	I			
SHZY25	0.064																			insH	N						V	I			
SHZY27	0.064																			insH	N		L		S		V	I			E
SHZY28	0.064																				N			V			V	I			
SHZY29	0.064	F	A																		del		L	T			V	I			
SHZY30	0.064	F	A																		del						V	I			
SHZY31	0.064																			insH	N		L		S		V	I			
SHZY32	0.064	F	A																	insH	N		L				V	I			
SHZY33	0.064																				del						V				
SHZY34	0.064		A																	insH	N		L		S		V	I			
SHZY35	0.064															T				insH	N		L	T	S		V	I			
SHZY36	0.064																			insH	N				S		V	I			
SHZY37	0.064															V					N				S		V				
SHZY39	0.064																								S		V				
SHZY40	0.064														Y					insH	N						V	I			
SHZY42	0.064	F																			N						V	I			
SHZY43	0.064				T																del			V			V	I			
SHZY45	0.064																										V	I			
SHZY46	0.064	F	A																	insH	N						V				
SHZY47	0.064	F	A																	insH	N		L		S		V	I			
SHZY48	0.064									R											del			V			V			G	
SHZY49	0.064		A											S		M				insH	N		L				V	I			
SHZY50	0.064	F	A																	insH	N			V	S		V	I			E
SHZY51	0.064	F	A																	insH	N						V				
Numbers of MR		3	7	3	2	0	0	0	1	1	1	1	1	3	0	2	1	1	1	8	13	0	2	3	3	1	4	10	0	0	1
Numbers of MS		16	19	0	2	1	1	1	1	1	1	0	2	1	1	5	0	0	1	34	42	1	11	9	15	0	46	30	1	1	3
P		0.514	0.759	0.01	0.23	1	1	1	1	0.415	0.415	0.233	0.556	0.036	1	0.66	0.233	0.233	0.415	0.338	1	1	0.713	1	0.52	0.233	1	0.756	1	1	1

Highlighted in yellow are strains that are resistant to amoxicillin.Highlighted green site is located within the PBP motif SXN/SXXK/KTG, and the remaining mutant sites are located in their nearby motifs.Numbers and capital letters located in the second and third rows indicate the position number and amino acid residues of strains 26695 PBP1A, PBP2 and PBP3.

MR: strains with point mutations and phenotypic resistance; MS: strains with point mutations but phenotypically sensitive.

Table S8. Mutations related to Tetracycline and furazolidone phenotypic resistance were previously described and first observed in this study

Gene	Mutation	Phenotypic TET-R		Phenotypic TET-S		P	Reference	Note
		MR	WR	MS	WS			
16S rRNA	AGA926-928TTC	Not detected					(40)	May lead to high levels of tetracycline resistance
	A928C	2	1	0	57	0.002	(41)	May lead to low levels of tetracycline resistance
	AG926-927GT	Not detected						
	A939C	Not detected					(42)	It may be related to tetracycline resistance
	A980G	1	2	0	57	0.05	This study	Mutation generates a stop lost
	G961A	1	2	0	57	0.05		Missense mutation
HP0399	A1378G	2	1	0	57	0.002		Encodes 30S ribosomal protein S1
Gene	Mutation					P	Reference	Note
		MR	WR	MS	WS			
oorD	A041G	Not detected					(43)	Possible association with furazolidone resistance from clinical isolates
	A122G							
	C349A（G）							
	A112G	5	0	50	5	1	(44)	A high frequency of these mutations has been detected, but whether they are associated with furazolidone resistance is unknown
	A335G	5	0	55	0	1		
	C156T	4	1	49	6	0.475		
	C165T	5	0	55	0	1		
porD	C347T/A/G	1	4	25	30	0.377		
	A346G	1	4	11	44	1		
	G353A	Not detected					(43)	Possible association with furazolidone resistance from clinical isolates
	A356G							
	C357T							

Table S9 Phenotypic and genotypic antibiotic resistance results of isolates

	CLA		LEV		AML		TET		MTZ		FR		MDR	
Strain number	G-R	P-R	G-R	P-R	G-R	P-R	G-R	P-R	G-R	P-R	G-R	P-R	G-R	P-R
SHZY01	R	R	S	S	R	R	R	R	S	R	S	S	R	R
SHZY02	S	S	R	R	R	S	S	S	R	R	S	S	S	S
SHZY03	S	S	S	S	R	S	S	S	R	R	S	S	S	S
SHZY04	R	R	R	R	R	R	S	S	R	R	S	S	R	R
SHZY05	S	R	R	R	R	S	S	S	S	R	S	S	R	R
SHZY06	S	R	S	S	R	R	S	S	S	R	S	S	R	R
SHZY07	R	R	R	R	R	S	S	S	S	R	S	S	S	R
SHZY08	S	S	R	R	R	S	S	S	S	R	S	S	S	S
SHZY09	S	S	S	S	S	S	S	S	S	R	S	S	R	S
SHZY10	S	S	S	S	S	S	S	S	S	R	S	S	S	S
SHZY11	R	R	S	S	R	S	S	S	S	R	S	S	S	S
SHZY12	S	S	R	R	R	S	S	S	S	R	S	R	R	R
SHZY13	R	R	S	R	R	S	S	S	R	R	S	S	R	R
SHZY14	R	R	S	S	R	S	S	S	S	R	S	R	R	R
SHZY15	R	R	R	R	S	S	S	S	S	R	S	S	S	R
SHZY16	S	S	S	S	S	S	S	S	S	R	S	S	S	S
SHZY17	R	R	S	S	R	S	S	S	S	R	S	R	R	R
SHZY18	R	R	R	R	S	S	S	S	S	R	S	S	S	R
SHZY19	S	S	R	R	R	S	S	S	R	R	S	S	S	S
SHZY20	S	S	S	S	R	S	S	S	S	R	S	S	S	S
SHZY21	R	R	S	S	R	S	S	S	R	R	S	S	S	S
SHZY22	R	R	S	S	R	S	S	S	R	R	S	R	R	R
SHZY23	S	R	R	R	R	S	S	S	S	R	S	S	R	R
SHZY24	R	R	S	S	S	S	S	S	S	R	S	R	R	R
SHZY25	S	S	R	R	S	S	S	S	S	R	S	S	S	S

SHZY26	S	S	R	R	S	R	S	S	S	R	S	S	R	R
SHZY27	S	S	R	R	S	S	S	S	S	R	S	S	S	S
SHZY28	R	R	S	S	S	S	S	S	S	S	S	S	S	S
SHZY29	R	R	R	R	R	S	S	S	R	R	S	S	S	R
SHZY30	R	R	S	S	R	S	S	S	R	R	S	S	S	S
SHZY31	R	R	R	S	S	S	S	S	S	R	S	S	S	S
SHZY32	S	S	S	S	R	S	S	S	S	S	S	S	S	S
SHZY33	S	S	S	S	S	S	S	S	S	R	S	S	R	S
SHZY34	R	R	S	S	R	S	S	S	R	R	S	S	R	S
SHZY35	S	S	R	R	R	S	S	S	S	R	S	S	S	S
SHZY36	S	S	S	S	S	S	S	S	S	R	S	S	S	S
SHZY37	S	S	S	S	R	S	S	S	R	R	S	S	S	S
SHZY38	S	S	R	R	R	S	S	S	S	R	S	S	S	S
SHZY39	S	S	R	R	S	S	S	S	S	R	S	S	S	S
SHZY40	S	S	S	S	R	S	S	S	S	R	S	S	S	S
SHZY41	R	R	R	R	R	R	R	R	S	R	S	S	R	R
SHZY42	S	S	S	S	R	S	S	S	S	R	S	S	S	S
SHZY43	R	R	R	R	R	S	S	S	R	R	S	S	R	R
SHZY44	R	R	R	R	R	S	S	S	S	R	S	S	S	R
SHZY45	S	S	S	S	S	S	S	S	S	R	S	S	S	S
SHZY46	R	R	S	R	R	S	S	S	S	R	S	S	R	R
SHZY47	R	R	S	R	R	S	S	S	S	R	S	S	R	R
SHZY48	R	R	R	R	S	S	S	S	R	R	S	S	R	R
SHZY49	R	R	R	S	R	S	S	S	S	R	S	S	S	S
SHZY50	S	S	S	S	R	S	S	S	R	R	S	S	S	S
SHZY51	S	S	S	S	R	S	S	S	S	S	S	S	S	S
SHZY52	R	R	R	R	R	R	S	R	R	R	S	S	R	R
SHZY53	R	R	R	R	R	R	S	S	S	S	S	S	R	R
SHZY54	S	S	S	S	R	R	S	S	S	S	S	S	R	S
SHZY55	S	S	R	R	R	R	S	S	S	R	S	S	S	R
SHZY56	S	R	S	S	R	R	S	S	S	R	S	S	R	R

SHZY57	S	S	R	R	S	R	S	S	R	R	S	S	R	R
SHZY58	S	S	S	S	R	R	S	S	S	R	S	S	S	S
SHZY59	S	S	S	S	R	R	S	S	S	S	S	S	S	S
SHZY60	R	R	R	R	R	R	S	S	R	R	S	S	R	R
c	45.924		41.676		1.775		39.310		2.632		/		7.556	
P	0.000		0.000		0.310		0.002		0.170		/		0.009	
Note:G-R:Genotypic resistance; P-R:Phenotypic resistance;G-R criterion for MDR is the occurrence of A149G mutation in fabH;P-R criterion for MDR is E-tset test result of ⩾3 antibiotic resistance														

Table S10 Quality metrics data for all sequenced genomes.

Samples	Coverage(X)	Contigs (>= 5000 bp)	Total length (>= 5000 bp)	ood Contigs (>= 500 bp	Largest contig	Total length	Gc (%)	N50	N75	L50	L75
SHZY01	365.22	21	1551021	34	413133	1573980	38.82	115353	53296	4	9
SHZY02	431.71	26	1566333	36	274193	1584053	38.72	68700	47213	7	14
SHZY03	349.84	26	1590162	40	193265	1603829	38.69	80334	51432	7	13
SHZY04	388.88	27	1529607	46	332543	1565163	38.85	69535	48705	6	13
SHZY05	319.88	22	1569993	39	330555	1594468	38.71	138260	49448	4	9
SHZY06	403.45	21	1541844	34	328836	1559360	38.86	110147	54462	4	9
SHZY07	232.61	41	1639833	2446	236745	4147903	39.52	2343	959	176	915
SHZY08	230.86	63	1782925	1815	199520	3696406	38.37	3917	1177	78	579
SHZY09	216.39	27	1672271	666	615399	2361143	38.31	64280	2303	7	59
SHZY10	345.34	24	1588355	124	229445	1693452	38.51	102224	41517	6	11
SHZY11	234.11	18	1601976	90	653009	1669619	38.6	106854	54759	3	8
SHZY12	174.84	30	1585371	788	277443	2250056	38.16	50526	2178	9	63
SHZY13	244.66	29	1617686	632	221361	2134883	38.25	56573	6438	8	27
SHZY14	372.29	25	1593710	2149	354807	3352918	39.4	3069	763	48	757
SHZY15	196.39	40	1616448	954	641228	2594884	38.37	29327	1551	15	186
SHZY16	309.88	30	1568539	687	239249	2076257	39.6	54881	6516	10	29
SHZY17	233.46	22	1584839	111	363182	1649444	38.86	107342	61404	5	10
SHZY18	107.35	56	1593512	7777	168501	8131123	39.58	1005	675	1728	4245
SHZY19	425.41	32	1671996	1714	255604	2984151	39.71	26983	805	18	482
SHZY20	269.11	77	1506130	1060	95793	2359536	39.27	14174	1253	39	199
SHZY21	333.89	35	1554315	87	193853	1609884	38.76	72715	35276	8	16
SHZY22	234.8	28	1659604	105	291820	1747656	38.39	105999	48656	6	12
SHZY23	221.16	21	1626267	566	653100	2111613	38.37	106202	11234	4	16
SHZY24	307.83	37	1670809	1569	333023	2886391	39.05	27945	840	19	401
SHZY25	276.64	24	1695247	62	371085	1729425	38.65	106470	52541	4	10
SHZY26	349.47	19	1587636	34	549291	1607168	38.69	107682	59761	3	8
SHZY27	362.22	28	1585070	40	229195	1604208	38.72	59689	42127	7	15
SHZY28	339.59	21	1623431	37	533227	1645683	38.66	107470	56348	4	9
SHZY29	359.67	16	1549400	29	332973	1566533	38.83	211325	64472	3	6
SHZY30	334.08	16	1566046	27	489905	1584501	38.73	174274	69069	3	7
SHZY31	369.31	20	1589338	32	419998	1609354	38.66	168670	59583	3	8
SHZY32	290.44	33	1531190	78	168341	1592804	38.9	66226	37105	7	15
SHZY33	276.22	25	1591142	38	240470	1616967	38.63	122206	52375	5	11
SHZY34	238.98	15	1592319	24	378052	1605822	38.71	158277	79547	3	7
SHZY35	352.51	21	1626393	35	275837	1643949	38.55	75945	57972	5	11
SHZY36	310.29	25	1572034	83	311817	1647922	38.74	104201	45535	5	12
SHZY37	380.33	20	1528158	33	466565	1547954	38.89	165933	56795	3	7
SHZY38	299.79	43	1697563	70	201594	1739821	38.54	61767	36105	9	17
SHZY39	370.89	31	1588684	51	160145	1635403	38.71	83920	42245	7	15
SHZY40	245.72	23	1580448	32	308322	1593939	38.66	98468	60834	5	10
SHZY41	302.91	36	1614687	83	249973	1672364	38.63	86501	41768	7	14
SHZY42	327.5	27	1573102	51	333220	1605927	38.72	93797	53618	5	11
SHZY43	288.35	19	1579207	75	318143	1643725	38.69	107436	63881	5	10
SHZY44	581	20	1541999	38	352473	1563887	38.84	181875	52881	3	9
SHZY45	647.46	20	1639179	34	213348	1658620	38.76	105784	54755	5	10
SHZY46	594.57	17	1613577	84	360199	1680948	38.77	146646	66517	4	7
SHZY47	688.03	22	1580613	46	358766	1617642	38.71	123422	69661	4	8
SHZY48	505.16	22	1638097	100	560012	1707488	38.67	110979	54176	4	9
SHZY49	444.48	31	1550265	73	213316	1611056	38.68	76502	35854	8	15
SHZY50	658.74	16	1552437	22	430132	1562930	38.85	115003	106976	4	7
SHZY51	495.63	21	1572998	33	347155	1595480	38.74	119798	53197	4	10
SHZY52	781.08	26	1543625	37	240948	1562435	38.85	75612	47228	5	11
SHZY53	655.97	28	1521966	101	314703	1588558	39.2	70171	47234	6	13
SHZY54	561.4	29	1611499	47	359622	1632347	38.63	102687	52723	4	11
SHZY55	690.31	28	1565253	580	302415	1937971	38.71	63750	20846	8	20
SHZY56	211.24	94	1603341	347	67568	2095753	38.53	14703	5539	36	89
SHZY57	751.43	27	1606565	45	204316	1622878	38.67	80104	49129	6	13
SHZY58	331.21	110	1587876	685	56303	2325260	38.45	10977	4042	54	145
SHZY59	764.04	25	1575531	305	234013	1896516	38.54	121811	34803	6	12
SHZY60	646.47	22	1582057	47	377491	1612294	38.75	110467	52293	4	10

Table S11 Phylogenetic tree genome information

PATRIC Genome Id	Total Genes	Single Copy	Name
210.6585	1366	916	Helicobacter pylori strain MHP31
869727.3	1390	914	Helicobacter pylori 908
210.6593	1390	916	Helicobacter pylori strain MHP12
210.6615	1392	916	Helicobacter pylori strain MHP13
85963.7	1412	916	Helicobacter pylori J99
210.4167	1434	916	Helicobacter pylori strain B31
907240.3	1436	915	Helicobacter pylori Gambia94/24
210.8509	1295	855	Helicobacter pylori SHZY56
1156914.3	1394	880	Helicobacter pylori NAB47
210.8463	1397	914	Helicobacter pylori SHZY41
1287061.3	1407	915	Helicobacter pylori UM018
1282880.3	1409	916	Helicobacter pylori NAK7
1055530.4	1410	916	Helicobacter pylori SNT49
210.8440	1423	915	Helicobacter pylori SHZY08
1355530.3	1430	916	Helicobacter pylori UM084
907238.3	1434	915	Helicobacter pylori India7
1355529.3	1435	914	Helicobacter pylori UM067
1191464.11	1453	916	Helicobacter pylori FD535
512562.5	1402	916	Helicobacter pylori Shi470
1163741.3	1406	913	Helicobacter pylori Shi169
1055529.4	1415	916	Helicobacter pylori Puno135
637913.3	1420	914	Helicobacter pylori v225d
1163739.3	1428	916	Helicobacter pylori Shi417
765963.4	1432	916	Helicobacter pylori PeCan4
765964.3	1449	916	Helicobacter pylori Cuz20
210.8483	1356	916	Helicobacter pylori SHZY55
210.8467	1363	909	Helicobacter pylori SHZY39
210.6579	1366	916	Helicobacter pylori strain MHP37
210.6613	1370	916	Helicobacter pylori strain MHP19

357544.13	1396	916	Helicobacter pylori HPAG1
210.6595	1401	916	Helicobacter pylori strain MHP11
210.8448	1410	916	Helicobacter pylori SHZY19
693745.3	1417	916	Helicobacter pylori B8
907237.3	1421	914	Helicobacter pylori Lithuania75
210.8468	1427	916	Helicobacter pylori SHZY38
1222003.3	1443	915	Helicobacter pylori Sahul64
1234600.3	1446	913	Helicobacter pylori Rif2
210.8507	1208	773	Helicobacter pylori SHZY58
210.8444	1283	859	Helicobacter pylori SHZY20
210.8429	1327	916	Helicobacter pylori SHZY04
210.8479	1331	912	Helicobacter pylori SHZY53
210.8466	1343	916	Helicobacter pylori SHZY37
210.8478	1346	916	Helicobacter pylori SHZY52
210.8451	1346	916	Helicobacter pylori SHZY30
210.8471	1349	916	Helicobacter pylori SHZY50
210.8472	1349	909	Helicobacter pylori SHZY49
210.8442	1350	916	Helicobacter pylori SHZY21
210.8469	1351	916	Helicobacter pylori SHZY44
210.8464	1352	915	Helicobacter pylori SHZY40
210.8452	1352	916	Helicobacter pylori SHZY29
210.8428	1352	916	Helicobacter pylori SHZY02
210.8465	1354	916	Helicobacter pylori SHZY42
210.8462	1355	916	Helicobacter pylori SHZY16
210.8482	1356	909	Helicobacter pylori SHZY59
210.8457	1356	912	Helicobacter pylori SHZY32
210.8437	1360	916	Helicobacter pylori SHZY06
210.8477	1361	916	Helicobacter pylori SHZY45
210.8435	1362	913	Helicobacter pylori SHZY12
210.8447	1363	904	Helicobacter pylori SHZY18
210.8427	1363	916	Helicobacter pylori SHZY01

210.8473	1365	916	Helicobacter pylori SHZY51
210.8476	1365	916	Helicobacter pylori SHZY47
210.8481	1366	916	Helicobacter pylori SHZY60
210.8439	1366	916	Helicobacter pylori SHZY05
210.8430	1368	916	Helicobacter pylori SHZY03
210.8460	1369	915	Helicobacter pylori SHZY36
210.8453	1370	916	Helicobacter pylori SHZY28
210.8470	1371	916	Helicobacter pylori SHZY43
210.8485	1375	916	Helicobacter pylori SHZY57
210.8456	1375	916	Helicobacter pylori SHZY31
210.845	1375	916	Helicobacter pylori SHZY27
210.8432	1376	916	Helicobacter pylori SHZY14
210.8458	1376	916	Helicobacter pylori SHZY34
210.8441	1377	916	Helicobacter pylori SHZY10
1355528.3	1378	914	Helicobacter pylori UM065
210.6606	1378	916	Helicobacter pylori strain MHP03
210.8454	1379	916	Helicobacter pylori SHZY26
210.8459	1380	916	Helicobacter pylori SHZY33
210.6600	1381	916	Helicobacter pylori strain MHP10
210.8475	1382	915	Helicobacter pylori SHZY48
210.8433	1382	916	Helicobacter pylori SHZY11
210.8461	1383	916	Helicobacter pylori SHZY35
210.8431	1383	912	Helicobacter pylori SHZY15
210.8438	1385	906	Helicobacter pylori SHZY07
210.8480	1386	916	Helicobacter pylori SHZY54
210.8455	1386	916	Helicobacter pylori SHZY25
210.8449	1386	916	Helicobacter pylori SHZY17
210.8443	1390	916	Helicobacter pylori SHZY23
210.8446	1392	904	Helicobacter pylori SHZY24
210.8474	1393	916	Helicobacter pylori SHZY46
210.8436	1394	915	Helicobacter pylori SHZY13

210.8445	1395	916	Helicobacter pylori SHZY22
290847.5	1395	916	Helicobacter pylori 51
102608.4	1401	916	Helicobacter pylori F32
1248726.3	1405	916	Helicobacter pylori OK310
210.8434	1410	916	Helicobacter pylori SHZY09
210.6605	1414	916	Helicobacter pylori strain MHP04
1352357.3	1406	912	Helicobacter pylori SouthAfrica50
1352356.3	1419	915	Helicobacter pylori SouthAfrica20
907239.3	1439	912	Helicobacter pylori SouthAfrica7

Table S12 Gene Family Statistics.

PGFam	Align_Score	Align_Length	Num_Seqs	Mean_Sqr_Freq	Prop_Gaps	Used_In_Analysis	Product
PGF_00029593	31.37	1028	100	0.979	0.001	TRUE	Multidrug efflux system, inner membrane proton/drug antiporter (RND type) => CmeF
PGF_03082131	30.09	979	100	0.962	0.005	TRUE	Uncharacterized membrane-anchored protein HP0586
PGF_05500127	28.57	874	100	0.966	0.003	TRUE	Valyl-tRNA synthetase (EC 6.1.1.9)
PGF_10357457	24.55	620	100	0.986	0.002	TRUE	Chaperone protein DnaK
PGF_01887544	24.51	690	100	0.933	0.022	TRUE	Phosphoglycerol transferase
PGF_04333086	24.49	662	100	0.952	0.009	TRUE	DNA ligase (NAD(+)) (EC 6.5.1.2)
PGF_00950554	24.17	676	100	0.93	0.041	TRUE	Excinuclease ABC subunit B
PGF_00022667	23.99	628	100	0.957	0.008	TRUE	Motility accessory factor
PGF_01960322	23.85	597	100	0.976	0.001	TRUE	Glutamine--fructose-6-phosphate aminotransferase [isomerizing] (EC 2.6.1.16)
PGF_05421852	23.4	649	100	0.919	0.05	TRUE	Chaperone protein HtpG
PGF_00982259	23.34	579	100	0.97	0.003	TRUE	Aspartyl-tRNA synthetase (EC 6.1.1.12) @ Aspartyl-tRNA(Asn) synthetase (EC 6.1.1.23)
PGF_00416129	22.93	538	100	0.988	0.001	TRUE	CTP synthase (EC 6.3.4.2)
PGF_10421966	22.8	550	100	0.972	0.003	TRUE	Dipeptide ABC transporter, substrate-binding protein DppA (TC 3.A.1.5.2)
PGF_00012987	22.61	524	100	0.988	0	TRUE	D-3-phosphoglycerate dehydrogenase (EC 1.1.1.95)
PGF_03192541	22.55	554	100	0.958	0.013	TRUE	Efflux ABC transporter, permease/ATP-binding protein
PGF_00022758	22.44	527	100	0.977	0.008	TRUE	Uncharacterized integral membrane protein HP0284
PGF_05877811	22.04	506	100	0.98	0.001	TRUE	AAA+ ATPase superfamily protein YifB/ComM, associated with DNA recombination
PGF_08028591	21.9	504	100	0.976	0.006	TRUE	Cardiolipin synthase (EC 2.7.8.-) phosphatidylethanolamine-utilizing, bacterial type ClsC
PGF_00420358	21.83	488	100	0.988	0	TRUE	Cytochrome c oxidase (cbb3-type) subunit CcoN (EC 1.9.3.1)
PGF_03316046	21.37	486	100	0.97	0	TRUE	Threonine synthase (EC 4.2.3.1)
PGF_00021217	21.34	492	100	0.962	0.002	TRUE	2,3-bisphosphoglycerate-independent phosphoglycerate mutase (EC 5.4.2.12)
PGF_00843497	21.25	501	100	0.949	0.017	TRUE	Cell division protein FtsA
PGF_00067554	20.94	475	100	0.961	0.008	TRUE	Aspartyl-tRNA(Asn) amidotransferase subunit B (EC 6.3.5.6) @ Glutamyl-tRNA(Gln) amidotransferase subunit B (EC 6.3.5.7)
PGF_00037219	20.85	487	100	0.945	0.016	TRUE	Multidrug efflux system, outer membrane factor lipoprotein => CmeD
PGF_00419628	20.8	457	100	0.973	0	TRUE	Coproporphyrinogen III oxidase, oxygen-independent (EC 1.3.99.22)
PGF_00018970	20.69	450	100	0.975	0.007	TRUE	Malate:quinone oxidoreductase (EC 1.1.5.4)
PGF_07396749	20.67	461	100	0.963	0.018	TRUE	Biotin carboxylase of acetyl-CoA carboxylase (EC 6.3.4.14)
PGF_02516909	20.34	426	100	0.986	0	TRUE	Enolase (EC 4.2.1.11)
PGF_00043007	20.09	441	100	0.956	0.011	TRUE	Putative periplasmic protein
PGF_01887428	19.63	435	100	0.941	0.021	TRUE	Uncharacterized protein HP_0971

PGF_10047030	19.51	409	100	0.965	0	TRUE	Adenosine deaminase (EC 3.5.4.4), alternative form
PGF_00043033	19.44	442	100	0.924	0.006	TRUE	Putative periplasmic protein
PGF_00063916	19.39	402	100	0.967	0.005	TRUE	Tyrosyl-tRNA synthetase (EC 6.1.1.1)
PGF_00416622	19.21	406	100	0.954	0.007	TRUE	Carboxynorspermidine decarboxylase (EC 4.1.1.96)
PGF_00046135	19.19	387	100	0.975	0.008	TRUE	Quinone-reactive Ni/Fe-hydrogenase small chain (EC 1.12.5.1)
PGF_06935032	18.97	411	100	0.936	0.018	TRUE	Adenylosuccinate synthetase (EC 6.3.4.4)
PGF_00019594	18.82	364	100	0.987	0.007	TRUE	Membrane protease family protein HP0248
PGF_05950073	18.74	380	100	0.961	0.005	TRUE	Cystathionine gamma-lyase (EC 4.4.1.1)
PGF_08932911	18.61	377	100	0.958	0	TRUE	Alanine racemase (EC 5.1.1.1)
PGF_08109371	18.47	374	100	0.955	0.008	TRUE	Membrane-bound lytic murein transglycosylase D
PGF_09077460	18.22	368	100	0.95	0.001	TRUE	1-deoxy-D-xylulose 5-phosphate reductoisomerase (EC 1.1.1.267)
PGF_02029783	18.22	364	100	0.955	0.011	TRUE	GTP-binding protein Obg
PGF_00420549	18.17	351	100	0.97	0.003	TRUE	Cytochrome c551 peroxidase (EC 1.11.1.5)
PGF_00822481	18.14	334	100	0.993	0	TRUE	Dipeptide ABC transporter, permease protein DppB (TC 3.A.1.5.2)
PGF_10025016	18.08	440	100	0.862	0.122	TRUE	Cysteine desulfurase (EC 2.8.1.7)
PGF_01888827	18.04	364	100	0.945	0.018	TRUE	nickel-cobalt-cadmium resistance protein (nccB)
PGF_00024234	18	398	100	0.903	0.034	TRUE	N-succinyl-L,L-diaminopimelate desuccinylase (EC 3.5.1.18)
PGF_00035074	17.98	350	100	0.961	0.006	TRUE	Possible RNA methyltransferase aq_898
PGF_00063951	17.98	386	100	0.915	0.029	TRUE	UDP-4-amino-4,6-dideoxy-N-acetyl-beta-L-altrosamine transaminase (EC 2.6.1.92)
PGF_00416620	17.97	347	100	0.964	0.006	TRUE	3'-to-5' oligoribonuclease B, Bacillus type
PGF_01888226	17.96	337	100	0.978	0	TRUE	hypothetical protein
PGF_01033770	17.87	351	100	0.954	0.001	TRUE	Dihydroorotate dehydrogenase (quinone) (EC 1.3.5.2)
PGF_04139053	17.8	339	100	0.967	0.005	TRUE	Uroporphyrinogen III decarboxylase (EC 4.1.1.37)
PGF_00015526	17.75	326	100	0.983	0	TRUE	Iron-sulfur cluster assembly scaffold protein IscU/NifU-like
PGF_00417658	17.68	343	100	0.955	0	TRUE	3-dehydroquinate synthase (EC 4.2.3.4)
PGF_01763778	17.41	328	100	0.961	0	TRUE	Lipid A biosynthesis lauroyl acyltransferase (EC 2.3.1.241)
PGF_01867249	17.4	339	100	0.945	0.009	TRUE	Putative periplasmic protein
PGF_00847718	17.32	339	100	0.941	0.027	TRUE	Aliphatic amidase AmiE (EC 3.5.1.4)
PGF_06655223	17.3	309	100	0.984	0	TRUE	Malonyl CoA-acyl carrier protein transacylase (EC 2.3.1.39)
PGF_01887000	17.24	333	100	0.944	0.009	TRUE	hypothetical protein
PGF_00017281	17.1	342	100	0.925	0.016	TRUE	Lipopolysaccharide core heptosyltransferase I
PGF_09566238	17.03	315	100	0.96	0.001	TRUE	Septum-associated rare lipoprotein A
PGF_01876949	16.9	314	100	0.954	0	TRUE	Keto-acid dehydrogenase
PGF_03815442	16.72	292	100	0.978	0	TRUE	Cytochrome c oxidase (cbb3-type) subunit CcoP (EC 1.9.3.1)
PGF_01889221	16.68	314	100	0.942	0.007	TRUE	3'-to-5' oligoribonuclease A, Bacillus type
PGF_00012356	16.65	293	100	0.973	0.007	TRUE	Homoserine kinase (EC 2.7.1.39)
PGF_00007027	16.58	302	100	0.954	0.011	TRUE	GTP-binding protein Era
PGF_00760084	16.46	305	100	0.943	0.005	TRUE	Cystathionine beta-synthase (EC 4.2.1.22)
PGF_01867431	16.28	277	100	0.978	0	TRUE	Sulfite exporter TauE/SafE family
PGF_02390924	16.24	326	100	0.9	0.055	TRUE	16S rRNA (cytosine(1402)-N(4))-methyltransferase (EC 2.1.1.199)

PGF_10160552	16.18	277	100	0.972	0.007	TRUE	Enoyl-[acyl-carrier-protein] reductase [NADH] (EC 1.3.1.9)
PGF_05366548	15.94	272	100	0.966	0.004	TRUE	Methionine ABC transporter substrate-binding protein
PGF_02450432	15.55	270	100	0.947	0.015	TRUE	Phosphatidate cytidylyltransferase (EC 2.7.7.41)
PGF_00067788	15.43	242	100	0.992	0	TRUE	[NiFe] hydrogenase nickel incorporation-associated protein HypB
PGF_05580933	15.32	278	100	0.919	0.015	TRUE	Peptide chain release factor N(5)-glutamine methyltransferase (EC 2.1.1.297)
PGF_01534078	15.24	331	100	0.838	0.121	TRUE	Murein endolytic transglycosylase MltG
PGF_08184693	15.22	287	100	0.899	0.05	TRUE	Heat-inducible transcription repressor HrcA
PGF_00832112	15.21	253	100	0.956	0.02	TRUE	Outer membrane protein HP_1469
PGF_01867887	15.11	263	100	0.932	0.03	TRUE	hypothetical protein
PGF_01888189	15.1	254	100	0.948	0.006	TRUE	hypothetical protein
PGF_00853991	15.06	278	100	0.904	0.028	TRUE	Diaminopimelate epimerase (EC 5.1.1.7)
PGF_00420363	14.98	232	100	0.983	0.001	TRUE	Cytochrome c oxidase (cbb3-type) subunit CcoO (EC 1.9.3.1)
PGF_03679540	14.95	258	100	0.931	0.022	TRUE	Urease accessory protein UreF
PGF_03095653	14.85	234	100	0.97	0.002	TRUE	Multidrug efflux system, membrane fusion component => CmeE
PGF_03790040	14.62	248	100	0.928	0.037	TRUE	Ribonuclease III (EC 3.1.26.3)
PGF_04574228	14.6	234	100	0.955	0	TRUE	Triosephosphate isomerase (EC 5.3.1.1)
PGF_08582746	14.56	227	100	0.966	0	TRUE	23S rRNA (guanosine(2251)-2'-O)-methyltransferase (EC 2.1.1.185)
PGF_10317621	14.53	244	100	0.93	0.017	TRUE	Integral membrane protein
PGF_00053048	14.52	237	100	0.943	0.026	TRUE	Aminodeoxyfutalosine nucleosidase (EC 3.2.2.30)
PGF_01889211	14.48	243	100	0.929	0.024	TRUE	hypothetical protein
PGF_01888947	14.41	312	100	0.816	0.136	TRUE	TolA protein
PGF_00055273	14.4	214	100	0.984	0.005	TRUE	Superoxide dismutase [Fe] (EC 1.15.1.1)
PGF_04498026	14.24	222	100	0.956	0	TRUE	Pyridoxal phosphate-containing protein YggS
PGF_10567705	14.18	223	100	0.949	0.027	TRUE	putative lipoprotein
PGF_00035591	14.09	289	100	0.829	0.159	TRUE	Predicted L-lactate dehydrogenase, Fe-S oxidoreductase subunit YkgE
PGF_12908847	14.07	218	100	0.953	0.004	TRUE	Endonuclease III (EC 4.2.99.18)
PGF_10420457	14.07	205	100	0.983	0	TRUE	MBL-fold metallo-hydrolase superfamily
PGF_06098642	14.02	208	100	0.972	0.005	TRUE	Thymidylate synthase ThyX (EC 2.1.1.148)
PGF_01871138	13.9	203	100	0.976	0	TRUE	hypothetical protein
PGF_01868963	13.68	280	100	0.817	0.101	TRUE	putative neuraminyllactose-binding hemagglutinin HpaA

Gene families are ranked by alignment score combining mean per-position variability, alignment length, and gappiness.

Reference

1. Megraud F. 2004. H pylori antibiotic resistance: prevalence, importance, and advances in testing. Gut 53:1374-84.
2. Hansomburana P, Anantapanpong S, Sirinthornpunya S, Chuengyong K, Rojborwonwittaya J. 2012. Prevalence of single nucleotide mutation in clarithromycin resistant gene of Helicobacter pylori: a 32-months prospective study by using hybridization real time polymerase chain reaction. J Med Assoc Thai 95 Suppl 3:S28-35.
3. Zhao LJ, Huang YQ, Chen BP, Mo XQ, Huang ZS, Huang XF, Wei LD, Wei HY, Chen YH, Tang HY, Huang GR, Qin YC, Li XH, Wang LY. 2014. Helicobacter pylori isolates from ethnic minority patients in Guangxi: resistance rates, mechanisms, and genotype. World J Gastroenterol 20:4761-70.
4. Caliskan R, Tokman HB, Erzin Y, Saribas S, Yuksel P, Bolek BK, Sevuk EO, Demirci M, Yilmazli O, Akgul O, Kalayci F, Cakan H, Salih B, Bal K, Kocazeybek B. 2015. Antimicrobial resistance of Helicobacter pylori strains to five antibiotics, including levofloxacin, in Northwestern Turkey. Rev Soc Bras Med Trop 48:278-84.
5. Phan TN, Santona A, Tran VH, Tran TN, Le VA, Cappuccinelli P, Rubino S, Paglietti B. 2015. High rate of levofloxacin resistance in a background of clarithromycin- and metronidazole-resistant Helicobacter pylori in Vietnam. Int J Antimicrob Agents 45:244-8.
6. Rimbara E, Noguchi N, Kawai T, Sasatsu M. 2008. Novel mutation in 23S rRNA that confers low-level resistance to clarithromycin in Helicobacter pylori. Antimicrob Agents Chemother 52:3465-6.
7. Khan R, Nahar S, Sultana J, Ahmad MM, Rahman M. 2004. T2182C mutation in 23S rRNA is associated with clarithromycin resistance in Helicobacter pylori isolates obtained in Bangladesh. Antimicrob Agents Chemother 48:3567-9.
8. Zerbetto De Palma G, Mendiondo N, Wonaga A, Viola L, Ibarra D, Campitelli E, Salim N, Corti R, Goldman C, Catalano M. 2017. Occurrence of Mutations in the Antimicrobial Target Genes Related to Levofloxacin, Clarithromycin, and Amoxicillin Resistance in Helicobacter pylori Isolates from Buenos Aires City. Microb Drug Resist 23:351-358.
9. Garrido L, Toledo H. 2007. Novel genotypes in Helicobacter pylori involving domain V of the 23S rRNA gene. Helicobacter 12:505-9.
10. Zhen-Hua Z, De-Qiang H, Yong X, Lin-Lin L, Nong-Hua L. 2013. Characterization of 23S rRNA gene mutation in primary and secondary clarithromycin-resistant Helicobacter pylori strains from East China. Turk J Gastroenterol 24:5-9.
11. Barile KA, Silva AL, Xavier JN, Assumpção MB, Corvelo TC. 2010. Characterization of 23S rRNA domain V mutations in gastric biopsy patients from the eastern Amazon. Mem Inst Oswaldo Cruz

105:314-7.

12. Rimbara E, Noguchi N, Kijima H, Yamaguchi T, Kawai T, Sasatsu M. 2007. Mutations in the 23S rRNA gene of clarithromycin-resistant Helicobacter pylori from Japan. Int J Antimicrob Agents 30:250-4.
13. Domanovich-Asor T, Motro Y, Khalfin B, Craddock HA, Peretz A, Moran-Gilad J. 2020. Genomic Analysis of Antimicrobial Resistance Genotype-to-Phenotype Agreement in Helicobacter pylori. Microorganisms 9.
14. Binh TT, Shiota S, Suzuki R, Matsuda M, Trang TT, Kwon DH, Iwatani S, Yamaoka Y. 2014. Discovery of novel mutations for clarithromycin resistance in Helicobacter pylori by using next-generation sequencing. J Antimicrob Chemother 69:1796-803.
15. Miftahussurur M, Shrestha PK, Subsomwong P, Sharma RP, Yamaoka Y. 2016. Emerging Helicobacter pylori levofloxacin resistance and novel genetic mutation in Nepal. BMC Microbiol 16:256.
16. Trespalacios AA, Rimbara E, Otero W, Reddy R, Graham DY. 2015. Improved allele-specific PCR assays for detection of clarithromycin and fluoroquinolone resistant of Helicobacter pylori in gastric biopsies: identification of N87I mutation in GyrA. Diagn Microbiol Infect Dis 81:251-5.
17. Miyachi H, Miki I, Aoyama N, Shirasaka D, Matsumoto Y, Toyoda M, Mitani T, Morita Y, Tamura T, Kinoshita S, Okano Y, Kumagai S, Kasuga M. 2006. Primary levofloxacin resistance and gyrA/B mutations among Helicobacter pylori in Japan. Helicobacter 11:243-9.
18. Seck A, Burucoa C, Dia D, Mbengue M, Onambele M, Raymond J, Breurec S. 2013. Primary antibiotic resistance and associated mechanisms in Helicobacter pylori isolates from Senegalese patients. Ann Clin Microbiol Antimicrob 12:3.
19. Teh X, Khosravi Y, Lee WC, Leow AH, Loke MF, Vadivelu J, Goh KL. 2014. Functional and molecular surveillance of Helicobacter pylori antibiotic resistance in Kuala Lumpur. PLoS One 9:e101481.
20. Tshibangu-Kabamba E, Yamaoka Y. 2021. Helicobacter pylori infection and antibiotic resistance - from biology to clinical implications. Nat Rev Gastroenterol Hepatol 18:613-629.
21. Rasheed F, Campbell BJ, Alfizah H, Varro A, Zahra R, Yamaoka Y, Pritchard DM. 2014. Analysis of clinical isolates of Helicobacter pylori in Pakistan reveals high degrees of pathogenicity and high frequencies of antibiotic resistance. Helicobacter 19:387-99.
22. Chu A, Wang D, Guo Q, Lv Z, Yuan Y, Gong Y. 2020. Molecular detection of H. pylori antibiotic-resistant genes and molecular docking analysis. Faseb j 34:610-618.
23. Jorgensen MA, Trend MA, Hazell SL, Mendz GL. 2001. Potential involvement of several

nitroreductases in metronidazole resistance in Helicobacter pylori. Arch Biochem Biophys 392:180-91.

24. Zhang S, Wang X, Wise MJ, He Y, Chen H, Liu A, Huang H, Young S, Tay CY, Marshall BJ, Li X, Chua EG. 2020. Mutations of Helicobacter pylori RdxA are mainly related to the phylogenetic origin of the strain and not to metronidazole resistance. J Antimicrob Chemother 75:3152-3155.

25. Binh TT, Suzuki R, Trang TT, Kwon DH, Yamaoka Y. 2015. Search for novel candidate mutations for metronidazole resistance in Helicobacter pylori using next-generation sequencing. Antimicrob Agents Chemother 59:2343-8.

26. Martínez-Júlvez M, Rojas AL, Olekhnovich I, Espinosa Angarica V, Hoffman PS, Sancho J. 2012. Structure of RdxA--an oxygen-insensitive nitroreductase essential for metronidazole activation in Helicobacter pylori. Febs j 279:4306-17.

27. Tanih NF, Ndip LM, Ndip RN. 2011. Characterisation of the genes encoding resistance to metronidazole (rdxA and frxA) and clarithromycin (the 23S-rRNA genes) in South African isolates of Helicobacter pylori. Ann Trop Med Parasitol 105:251-9.

28. Hu Y, Zhang M, Lu B, Dai J. 2016. Helicobacter pylori and Antibiotic Resistance, A Continuing and Intractable Problem. Helicobacter 21:349-63.

29. Gong Y, Yuan Y. 2018. Resistance mechanisms of Helicobacter pylori and its dual target precise therapy. Crit Rev Microbiol 44:371-392.

30. Butlop TR, Mungkote NT, Chaichanawongsaroj NT. 2016. Analysis of allelic variants of rdxA associated with metronidazole resistance in Helicobacter pylori: detection of common genotypes in rdxA by multiplex allele-specific polymerase chain reaction. Genet Mol Res 15.

31. Chua EG, Debowski AW, Webberley KM, Peters F, Lamichhane B, Loke MF, Vadivelu J, Tay CY, Marshall BJ, Wise MJ. 2019. Analysis of core protein clusters identifies candidate variable sites conferring metronidazole resistance in Helicobacter pylori. Gastroenterol Rep (Oxf) 7:42-49.

32. Tsugawa H, Suzuki H, Satoh K, Hirata K, Matsuzaki J, Saito Y, Suematsu M, Hibi T. 2011. Two amino acids mutation of ferric uptake regulator determines Helicobacter pylori resistance to metronidazole. Antioxid Redox Signal 14:15-23.

33. Chang KC, Ho SW, Yang JC, Wang JT. 1997. Isolation of a genetic locus associated with metronidazole resistance in Helicobacter pylori. Biochem Biophys Res Commun 236:785-8.

34. Albert TJ, Dailidiene D, Dailide G, Norton JE, Kalia A, Richmond TA, Molla M, Singh J, Green RD, Berg DE. 2005. Mutation discovery in bacterial genomes: metronidazole resistance in Helicobacter pylori. Nat Methods 2:951-3.

35. Tshibangu-Kabamba E, Ngoma-Kisoko PJ, Tuan VP, Matsumoto T, Akada J, Kido Y, Tshimpi-Wola

A, Tshiamala-Kashala P, Ahuka-Mundeke S, Ngoy DM, Disashi-Tumba G, Yamaoka Y. 2020. Next-Generation Sequencing of the Whole Bacterial Genome for Tracking Molecular Insight into the Broad-Spectrum Antimicrobial Resistance of Helicobacter pylori Clinical Isolates from the Democratic Republic of Congo. Microorganisms 8.

36. Kwon YH, Kim JY, Kim N, Park JH, Nam RH, Lee SM, Kim JW, Kim JM, Park JY, Lee DH. 2017. Specific mutations of penicillin-binding protein 1A in 77 clinically acquired amoxicillin-resistant Helicobacter pylori strains in comparison with 77 amoxicillin-susceptible strains. Helicobacter 22.
37. Tshibangu-Kabamba E, Yamaoka Y. 2021. Helicobacter pylori infection and antibiotic resistance - from biology to clinical implications. Nat Rev Gastroenterol Hepatol doi:10.1038/s41575-021-00449-x.
38. Rimbara E, Noguchi N, Kawai T, Sasatsu M. 2008. Mutations in penicillin-binding proteins 1, 2 and 3 are responsible for amoxicillin resistance in Helicobacter pylori. J Antimicrob Chemother 61:995-8.
39. Qureshi NN, Gallaher B, Schiller NL. 2014. Evolution of amoxicillin resistance of Helicobacter pylori in vitro: characterization of resistance mechanisms. Microb Drug Resist 20:509-16.
40. Glocker E, Berning M, Gerrits MM, Kusters JG, Kist M. 2005. Real-time PCR screening for 16S rRNA mutations associated with resistance to tetracycline in Helicobacter pylori. Antimicrob Agents Chemother 49:3166-70.
41. Toledo H, López-Solís R. 2010. Tetracycline resistance in Chilean clinical isolates of Helicobacter pylori. J Antimicrob Chemother 65:470-3.
42. Dadashzadeh K, Milani M, Rahmati M, Akbarzadeh A. 2014. Real-time PCR detection of 16S rRNA novel mutations associated with Helicobacter pylori tetracycline resistance in Iran. Asian Pac J Cancer Prev 15:8883-6.
43. Zamani M, Rahbar A, Shokri-Shirvani J. 2017. Resistance of Helicobacter pylori to furazolidone and levofloxacin: A viewpoint. World J Gastroenterol 23:6920-6922.
44. Dong F, Ji D, Huang R, Zhang F, Huang Y, Xiang P, Kong M, Nan L, Zeng X, Wu Y, Bao Z. 2015. Multiple Genetic Analysis System-Based Antibiotic Susceptibility Testing in Helicobacter pylori and High Eradication Rate With Phenotypic Resistance-Guided Quadruple Therapy. Medicine (Baltimore) 94:e2056.
